# The role of affective learning biases in daily food behaviour

**DOI:** 10.1038/s44271-026-00511-x

**Published:** 2026-07-24

**Authors:** Malika Tapparel, Hugo Najberg, Lucas Spierer

**Affiliations:** Laboratory for Neurorehabilitation Science, Medicine Section, Faculty of Science and Medicine, University of Fribourg, 1700 Fribourg, Switzerland

**Keywords:** Medical research, Risk factors, Biomarkers

## Abstract

Cravings contribute to eating behaviours and health, yet how they emerge and translate into consumption remains unclear. Individual biases in how we learn to assign value to cues and actions, through Pavlovian conditioning (sign-tracking and goal-tracking) and reinforcement learning (model-free and model-based), provide a framework for exploring these behaviours. Cravings, emerging from associations between environmental cues and physiological responses, may be amplified by learning biases enhancing the cues’ salience (i.e., sign-tracking bias) and habitual behaviour (i.e., model-free). Learning biases may also moderate the relationship between cravings and consumption by intensifying automatic and habitual processes. Lastly, approach bias, which measures automatic processes involved in craving formation, may be associated with learning biases. To test these hypotheses, we conducted a two-week longitudinal study in 81 participants (minimum post-exclusion sample of 65) using ecological momentary assessments via a smartphone-based app to measure high-caloric food cravings, food intake, and approach-avoidance tendencies in daily life. Learning biases were assessed using Pavlovian conditioning and sequential learning tasks. Results indicate that sign-tracking bias was not related to craving intensity or variability, whereas model-free bias was associated with higher mean cravings but not lower day-to-day craving variability. The hypothesis linking cravings to consumption was not tested due to a consumption floor effect, and neither learning bias was associated with approach bias. The healthy-weight sample may explain our null findings, as they may show limited expression of high-caloric food cravings and consumption. Overall, these findings suggest that reinforcement learning tendencies may contribute to everyday craving dynamics.

## Abstract (web summary)

How individuals learn to assign value to cues and actions may help explain how eating behaviours emerge. This study investigated whether reinforcement learning biases are associated with food cravings, consumption, and approach tendencies in daily life.

## Introduction

Food cravings are defined as strong desires to eat specific foods^1^, typically energetically dense and palatable items^2–4^. While cue-induced cravings influence eating habits^2,4–9^, how they emerge and interact with consumption remains unclear.

Learning mechanisms in Pavlovian and reinforcement learning explain how environmental cues gradually acquire motivational value and, in turn, influence consumption choices^10^. In Pavlovian learning^11^, palatable food items (and the related cues) become associated with both central (e.g., brain reward circuitry) and peripheral (e.g., salivation, digestive readiness) responses. These responses may reflect pleasure-related physiological reactions and preparatory mechanisms for food consumption. In reinforcement learning, values are attributed to actions in response to food cues^12^. Biases in those learning mechanisms represent a relevant framework to understand cravings and their link to consumption^10^ because they influence how people respond to food cues, ultimately affecting eating behaviours. In Pavlovian learning, individuals biased toward sign-tracking attribute incentive value to the cue itself, and goal-trackers focus on the cue-outcome predictive relationship^13,14^. Attentional bias towards food cues - an indirect measure of cues’ incentive salience in sign-trackers - has been consistently linked with tendencies for binge eating and food cravings^15^. In reinforcement learning, individuals who are biased toward model-free (habitual) learning update action values directly from reward prediction errors. For example, their favourite coffee would be associated with a positive value due to consistently rewarding, coffee-related past experiences. In contrast, model-based (goal-directed) learners build internal models of the environment to predict outcomes. For example, they might estimate the value of different coffees by considering factors like cost, time, and health benefits^16,17^. A greater reliance on model-free learning has been reported in patients with obesity^18^ and binge eating disorders^19^ compared to healthy controls.

Building on the existing literature demonstrating that environmental food cue valuation and food-related decision-making are shaped by Pavlovian and reinforcement-based mechanisms, we aim to investigate whether and how these learning processes influence cravings and consumption behaviours. To achieve this, we examine the following theoretical hypotheses:

Firstly, we hypothesised that learning biases are positively associated with cravings. Food cravings emerge through associative learning, where environmental cues (e.g., the sight of food packaging) become conditioned stimuli. These stimuli trigger physiological responses such as increased salivation, heart rate, and gastric activity, which are consciously perceived as cravings^1,6,20–22^. Sign-trackers, who assign higher incentive salience to conditioned stimuli like food cues^10,13,14^, may exhibit stronger or faster conditioning and, in turn, cravings in response to similar environmental cues^10,23^. Additionally, model-free learning biases may indirectly contribute to elevated cravings by reinforcing habitual behaviours and intensifying physiological responses to food cues. For instance, habitual model-free behaviours, such as consistently consuming chocolate after lunch, can elicit enhanced cephalic phase responses (CPRs), which are conditioned anticipatory physiological reactions (e.g., salivation, gastric activity) that precede nutrient absorption^24^. These heightened CPRs amplify the conscious perception of cravings. Additionally, model-free learning updates action values based on reward prediction errors, continuously reinforcing the perceived value of craved items. This cycle strengthens the association between environmental cues and cravings, further embedding the habitual craving response in a feedback loop that perpetuates itself.

Secondly, we hypothesised that learning biases positively moderate the relationship between cravings and food consumption. Affective learning plays a pivotal role in how cravings translate into consumption behaviours through three primary mechanisms: (i) automatic responses to specific stimuli, manifested in preparatory behaviours such as approaching cues that predict food availability (e.g., a restaurant sign) or habitual food choices^25^; (ii) habitual control, exemplified by routines like drinking coffee after lunch^26^; and (iii) goal-directed, model-based control, which facilitates forward-looking decisions, such as avoiding foods associated with long-term health risks^26^.

Sign-trackers and model-free learners exhibit stronger Pavlovian and habitual control, making them more susceptible to impulsive and compulsive traits—key behavioural drivers of food intake^10,19,27–29^. Compared to goal-trackers, sign-trackers display heightened bottom-up reward signalling and diminished top-down cognitive control^10,30^. These characteristics amplify the impact of cravings on their consumption behaviours, as affective learning reinforces the automatic and habitual components of their decision-making.

Food cravings may exacerbate the competition between behavioural controllers by disproportionately activating Pavlovian and habitual systems, potentially overriding goal-directed control. This dynamic is particularly pronounced in individuals with sign-tracking and model-free biases, who may exhibit increased consumption of palatable foods compared to goal-tracking and model-based learners under similar craving conditions. Such biases not only intensify the translation of cravings into consumption but also highlight the moderating role of learning styles in shaping food-related behaviours.

Lastly, we hypothesised that learning biases are positively associated with approach bias. Approach bias is defined as the tendency to respond more quickly when approaching rather than avoiding food stimuli and is thought to originate in part from automatic processes^31^. In the context of eating behaviour, approach bias is a direct measure of the automatic processes involved in craving formation and is assumed to drive consumption behaviour^31^, it may thus be positively associated with sign-tracking bias as they both capture automatic processes. Additionally, model-free bias may be associated with approach bias through the intensification of physiological responses to food cues.

**Fig. 1:**
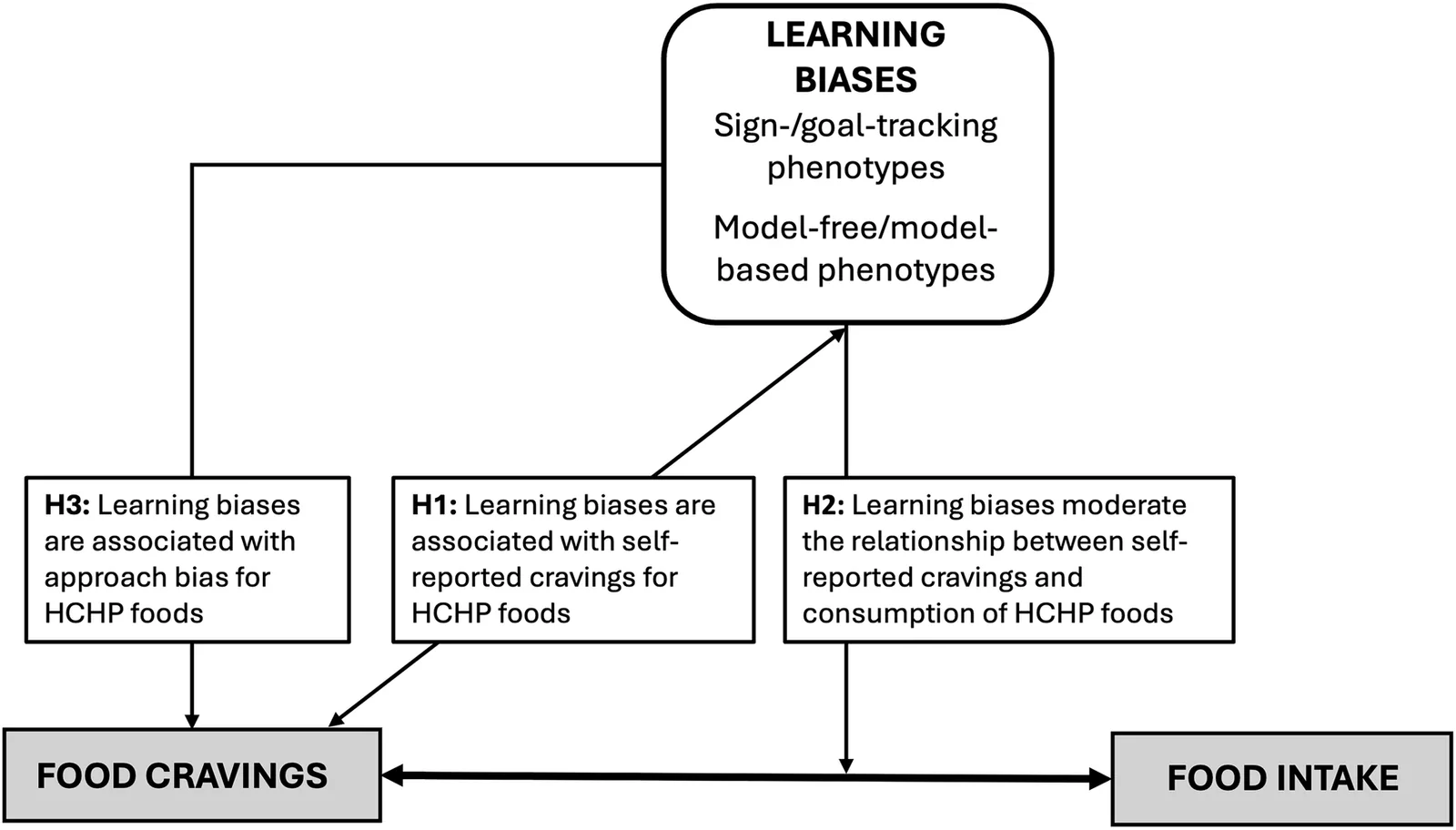
Hypothesised mechanisms of the relationship between learning biases, food cravings, and food intake. Learning biases are expressed as sign-/goal-tracking phenotypes and model-free/model-based phenotypes. H1: Learning biases are associated with self-reported cravings for highly caloric, highly processed (HCHP) foods. H2: Learning biases moderate the relationship between self-reported cravings and HCHP food intake. H3: Learning biases are associated with approach bias for HCHP foods.

Based on our theoretical hypothesis, we tested whether:

- H1) Pavlovian/reinforcement learning biases are correlated with self-reported cravings for high-caloric highly palatable (HCHP) foods (Fig. 1, Table 1; H1). We tested this hypothesis by analysing the correlation between Pavlovian bias and self-reported cravings (H1a). We then analysed the correlation between reinforcement learning bias and self-reported cravings (H1b). The hypothesised mechanisms of action stated above lead us to expect an effect between cravings and sign-tracking, which might not be observed between cravings and reinforcement learning bias.

**Table 1:** Design Table

Question	Hypothesis	Sampling plan	Analysis Plan	Interpretation given to different outcomes
Are learning biases positively correlated with self-reported cravings?	H1a: Pavlovian bias is positively correlated with self-reported cravings for HCHP foods	63 participants based on a priori power analysis with an r of 0.4, alpha of 0.05 and power of 0.95 Were planned to be excluded during analyses: - participants with an error rate above 10% at the Forced Choice task	Two correlations with 1) mean of daily self-reported HCHP food cravings, and 2) standard deviation of daily self-reported HCHP food cravings for both (H1a: regression coefficient of CS value on the eye-gaze index for Pavlovian Learning; H1b: w index for SL) with an alpha of 0.025 to account for multiple testing.	If *p* < 0.025 (Bonferroni-adjusted significance level) on at least one correlation, then the Pavlovian bias is significantly associated with elevated cravings for HCHP foods. A mean difference would suggest a shift in craving intensity. A standard deviation difference would suggest a shift in craving variability. If *p* ≥ 0.025, we will conclude that we cannot reject the null hypothesis for H1a.
	H1b: model-free bias is positively correlated with self-reported cravings for HCHP foods	63 participants based on a priori power analysis with an r of 0.4, alpha of 0.05 and power of 0.95 Were planned to be excluded during analyses: - participants with more than 20% of excluded trials - participants with a probability of stay when common rewarded trial worse than chance		If *p* < 0.025 (Bonferroni-adjusted significance level) on at least one of the outcomes of self-reported cravings and the reinforcement learning bias (w), then the reinforcement learning bias is significantly associated with elevated cravings for HCHP foods. A mean difference would suggest a shift in craving intensity. A standard deviation difference would suggest a shift in craving variability. If *p* ≥ 0.025, we will conclude that we cannot reject the null hypothesis for H1b.
Is there a positive moderating effect of learning biases on the relationship between self-reported cravings and consumption of HCHP foods	H2a: Pavlovian bias moderates positively the relationship between self-reported cravings and consumption of HCHP foods	76 participants based on a priori power analysis with an R^2^ of 0.16, an alpha of 0.05 and a power of 0.95 Exclusion criteria are the same as above.	A linear regression analysis with the predictors craving, learning bias (H2a: regression coefficient of CS value on the eye-gaze index for Pavlovian Learning; H2b: w index for SL), and their interaction on the outcome variable consumption of HCHP foods, using the lmer() function in R.	If *p* < 0.05 on the interaction term of cravings and the Pavlovian bias, then we conclude that the Pavlovian bias has a moderating effect on the relationship between self-reported cravings and consumption of HCHP foods. This would indicate that conditioned responses to food-associated cues amplify the impact of cravings on consumption. Such an effect would support the idea that automatic, cue-driven behaviours shape eating habits. If *p* ≥ 0.05, we will conclude that we cannot reject the null hypothesis for H2a.
	H2b: Reinforcement learning bias moderates positively the relationship between self-reported cravings and consumption of HCHP foods			If *p* < 0.05 on the interaction term of cravings and reinforcement learning bias, then we conclude that the reinforcement learning bias has a moderating effect on the relationship between self-reported cravings and consumption of HCHP foods. This would suggest that individuals who rely more on model-free learning are more likely to translate cravings into habitual food choices. If *p* ≥ 0.05, we will conclude that we cannot reject the null hypothesis for H2b.
Are learning biases positively correlated with approach bias?	H3a: Pavlovian bias is positively correlated with approach bias for HCHP foods	63 participants based on a priori power analysis with an r of 0.4, alpha of 0.05 and power of 0.95 Were planned to be excluded during analyses: - participants with an error rate above 10% at the Forced Choice task	Two correlations with 1) mean of approach biases for HCHP foods, and 2) standard deviation of approach biases for HCHP foods for both (H3a: regression coefficient of CS value on the eye-gaze index for Pavlovian Learning; H3b: w index for SL) with an alpha of 0.025 to account for multiple testing.	If *p* < 0.025 (Bonferroni-adjusted significance level) on at least one correlation, then the Pavlovian bias is significantly associated with higher approach bias for HCHP foods. A mean difference would suggest that individuals with stronger Pavlovian biases tend to exhibit a generally higher tendency to approach HCHP foods, indicating a shift in overall approach bias. A difference in standard deviation would suggest that Pavlovian biases are associated with greater variability in approach behaviour towards HCHP foods. If *p* ≥ 0.025, we will conclude that we cannot reject the null hypothesis for H3a.
	H3b: model-free bias is positively correlated with approach bias for HCHP foods	63 participants based on a priori power analysis with an r of 0.4, alpha of 0.05 and power of 0.95 Were planned to be excluded during analyses: - participants with more than 20% of excluded trials - participants with a probability of stay when common rewarded trial worse than chance		If *p* < 0.025 (Bonferroni-adjusted significance level) on at least one correlation, then the reinforcement learning bias is significantly associated with higher approach bias for HCHP foods. A mean difference would suggest that model-free bias directly or indirectly contributes to a higher overall tendency to approach HCHP foods. A standard deviation difference would suggest that model-free bias directly or indirectly influences the variability in approach behaviour. If *p* ≥ 0.025, we will conclude that we cannot reject the null hypothesis for H3b.
- H2) Pavlovian/reinforcement learning biases moderate the relationship between self-reported cravings and consumption (i.e., self-reported consumption) of HCHP foods (Fig. 1, Table 1; H2). We tested this hypothesis by examining Pavlovian bias (H2a) and reinforcement learning bias (H2b) as the moderating factors.
- H3) Pavlovian/reinforcement learning biases are correlated with approach bias for HCHP foods (Fig. 1, Table 1; H3). We tested this hypothesis by analysing the correlation between Pavlovian bias and approach bias (H3a). We then analysed the correlation between reinforcement learning bias and approach bias (H3b). The hypothesised mechanisms of action stated above lead us to expect an effect between approach bias and sign-tracking, which might not be observed between approach bias and reinforcement learning bias. Moreover, we used a descriptive approach to test whether Pavlovian/reinforcement learning biases are more strongly correlated with either approach bias or self-reported cravings for HCHP foods, comparing the correlation between learning biases and these two measures (i.e., self-reported cravings and approach bias, respectively).

We addressed these questions with a two-week longitudinal observational study using Ecological Momentary Assessments (EMA) to measure HCHP-induced self-reported cravings and consumption. The EMA approach involved collecting data in real-time and in participants’ natural environments, enhancing ecological validity and minimising response bias and confounds related to retrospective memory deformations^4,32,33^. This approach has been shown to be an effective method for capturing the specificity, intensity, and diversity of food cravings and consumption in real time, providing insights that would not be possible through retrospective measures^4,34^. To measure approach bias, we used a mobile approach-avoidance task (mobile-AAT)^35,36^ with repeated measures conducted over the two weeks. This method improves the reliability issues commonly associated with single-measure implicit assessments, such as temporal fluctuations in bias scores and their covariation with factors like affect or hunger^36^. The Pavlovian bias is indexed as the regression coefficient of a linear regression predicting the gaze direction from the reward value during a Pavlovian conditioning task^37^. To evaluate reinforcement learning bias, we used the weighting parameter *w* indexing the relative contribution of model-free and model-based systems in the net value of the action in a sequential learning task^19,38^. The use of Pavlovian and sequential learning tasks is well-established in the literature^19,27,37,38^, and those tasks are recognised as the standard approach for capturing learning biases.

## Methods

### Ethics information

All procedures were approved by the *Commission cantonale (VD) d’éthique de la recherche sur l’être humain* (CER-VD; #2025-00811) and performed in accordance with the relevant guidelines and regulations, as well as the Declaration of Helsinki.

### Protocol Registration

The Stage 1 protocol for this Registered Report was accepted in principle on June 20^th^, 2025. The protocol, as accepted by the journal, can be found at https://doi.org/10.6084/m9.figshare.29445518.

### Design

#### Pilot analysis

We conducted a pilot analysis to assess the feasibility and interpretability of the measures used in this study, which can be found in the Supplementary Material (Section 4).

#### Recruitment and screening

The participants were recruited via public advertisement.

The inclusion criteria included 18 to 65-year-old healthy individuals with a body mass index (BMI) between 18.5 and 28. The exclusion criteria were current or planned diet, medical conditions that could affect eating behaviour, pregnancy, and visual problems disallowing the completion of computerised tasks without glasses. Additionally, we invited women to start the study during their menstrual cycle (early follicular phase) to ensure hormonal consistency during the study period.

Although our effects of interest are theoretically not influenced by gender, we aimed to recruit a gender-balanced sample to ensure the generalisability of our results (50% males and 50% females) with a ± 20% deviation range. If recruitment deviated from this target, we planned to continue recruiting participants from the underrepresented group until the specified gender balance was achieved.

#### Study procedure

Participants accessed an information sheet and gave their informed consent online, as well as filled out a custom-made general health questionnaire for eligibility screening.

After the eligibility screening, participants were invited to come to our laboratory at the University of Fribourg to complete the sequential learning (SL) and Pavlovian conditioning tasks.

All the following cravings and consumption questionnaires, as well as the mobile approach-avoidance task (mobile-AAT), were completed in a homemade Android smartphone application. Participants were extensively instructed about the smartphone app, Ecological Momentary Assessments (EMA) and mobile-AAT procedure. Participants who did not have an Android device were lent a smartphone.

During the following two weeks, participants filled out six daily EMA questionnaires about their self-reported cravings and consumption, as well as the mobile-AAT task every other day.

Participants were compensated with a monetary reward, and participants who completed less than 50% of the EMA questionnaires were excluded from the study and compensated pro rata temporis.

#### Stimuli

For the self-reported cravings and consumption questionnaire, built-in icons from Microsoft PowerPoint and web-sourced icons were used (see Supplementary Material; Section 1.3).

For the mobile-AAT images, 129 food images and 16 object images selected from the Food-pics^39^ database were used. The list of selected images can be downloaded from our OSF study page (see Data Availability Statement section).

For the SL and Pavlovian conditioning task, the character images and the fractals were generated using AI tools (see Supplementary Material; Section 1.1 and 1.2).

#### Custom-made health questionnaire

To verify the eligibility criteria, a custom-made health questionnaire was used to assess the participants’ age and BMI, general health, and visual impairments.

#### EMA of self-reported cravings and consumption

The procedure was adapted from Roefs et al.^4^.

Participants received six daily notifications over two weeks, prompting them to open the app and complete both the self-reported cravings and consumption questionnaires for high-caloric highly palatable (HCHP) food items. These prompts were evenly distributed across waking hours, with one notification occurring approximately every 2.5 hours. Participants could customise their waking and sleeping times within the app at the beginning of the experiment.

For both questionnaires, participants were presented with 20 icons representing the main 19 food categories in Switzerland, as well as a “more” icon displaying a plus sign for participants to manually enter additional food items not represented in the categories.

Food items entered via the “more” icon were reclassified into HCHP, other, or non-food rather than on the exact category label. The categorisation procedure was developed *ad hoc* and was not preregistered.

Categorisation was performed independently by three researchers. Items that did not achieve a two-thirds agreement were further discussed until a final classification was reached. Across the 471 “more” items, 79.2% achieved full consensus, 18.1% reached a two-out-of-three agreement, and 2.7% required discussion. Details on the categorisation procedure can be found in the Supplementary Material (Section 5).

##### Self-reported cravings questionnaire

Upon receiving a notification, participants were prompted to open the app and complete the self-reported cravings questionnaire. Participants first answered the question, “How strong is your craving to eat?” Note that in French, the term “craving” does not have a direct equivalent and was defined as a “strong desire to eat”. Participants responded using a visual analogue scale ranging from “not at all” to “very much”. Participants then identified the type of food they desire with the question “Which one of the following foods do you crave?” Participants could select one or more food categories represented by the food icons (see Table 2) or choose “No specific food” if applicable. Finally, participants rated the intensity of their craving for the selected food item(s) by answering, “How strong do you crave an item from this category?” with the corresponding food icon displayed alongside the question and a visual analogue scale ranging from “not at all” to “very much”. Daily self-reported cravings were computed as the number of HCHP food cravings multiplied by the intensity of the cravings (ranging from 0 to 1), divided by the total number of daily assessments.

**Table 2:** Food icons within each food category

Food category	Represented by icons
HCHP	Chocolate, biscuit, salted snack, candy, pastry, cheese, cake, hamburger, pizza, iced dessert
Staple	Cereal, bread & sandwiches, rice dish & potato side, pasta, soup
Greens	Fruit, vegetable
Proteins	Meat, fish

##### Self-reported consumption questionnaire

Upon receiving a notification, participants were prompted to open the app and complete the self-reported consumption questionnaire. Participants first answered the question, “Did you eat anything since the last assessment?” by selecting either “Yes” or “No”. If they selected “Yes”, they then identified the type of food they consumed by answering the question “Which one of the following foods did you eat?” and selected one or more food categories represented by the food icons (Table 2). Finally, participants reported the quantity of food consumed by answering “How many servings of food from this category did you have?” This question displayed the corresponding food icon alongside a drop-down menu, allowing participants to select the number of servings. Participants received extensive instructions about the definition of servings for each category at the beginning of the study. Daily self-reported consumption was computed as the number of HCHP food consumption events, defined as eating events within a category that exceeded a minimum of 200 kcal, a threshold reflecting a typical snacking event for HCHP foods (e.g., 1 croissant ≈ 230 kcal, 1 small ice cream cup ≈ 200 kcal).

#### Mobile approach-avoidance task (Mobile-AAT)

To assess food approach bias in daily life, we used a homemade mobile approach-avoidance task (mobile-AAT) adapted from the version developed by Zech et al. (2023), with the procedure based on^36^. Participants received a notification prompting them to complete the mobile-AAT task every two days, 1 hour before dinner time, resulting in 7 individual assessments. Participants could customise their usual dinner time at the beginning of the experiment.

##### Analogue scales of explicit liking

To personalise food items in the mobile-AAT, participants first rated their explicit liking of the food images on a visual analogue scale. Food images were presented in a random sequence, and participants rated them on a scale from 0 (“not at all”) to 100 (“very much”) in response to the question, “Imagine eating this, how much do you like it?” Only the 12 most liked food items were included in the mobile-AAT. Control stimuli consisted of neutral, non-food objects.

##### Mobile-AAT

Participants received a notification once every two days to perform the task on their smartphones, held horizontally. Participants approached stimuli by rapidly moving their phone towards themselves and avoided stimuli by moving their phone away, following the on-screen instructions (e.g., “Move the food towards you and the objects away from you”).

The AAT consists of four blocks per session. Each block included individualised food images and control object stimuli. Before each block, participants completed four practice trials using non-personalised stimuli to familiarise themselves with the task instruction. Instructions alternated between blocks, with participants instructed to “avoid foods and approach objects” in the first blocks and to “approach foods and avoid objects” in the second blocks. Each stimulus was displayed once per block, to be approached and avoided equally often. One session included 96 trials and lasted about 6 minutes.

Each trial started with a 1.5 s fixation dot followed by the stimulus presentation. Incorrect responses were followed by an error cross and omissions of response ( > 2 s) were followed by a clock icon, correct responses were followed immediately by the next trial.

The reaction time (RT) was measured as the time from picture to movement onset. The approach bias for HCHP food was computed as the difference between reaction times (RTs) for HCHP food avoidance and approach trials, minus the difference between RTs for object avoidance and approach trials.

#### Pavlovian conditioning task

Participants completed a Pavlovian conditioning task^37^ to assess their Pavlovian bias, followed by a forced choice task. The method is replicated from our previous work^40^.

##### Pavlovian conditioning task

**Fig. 2:**
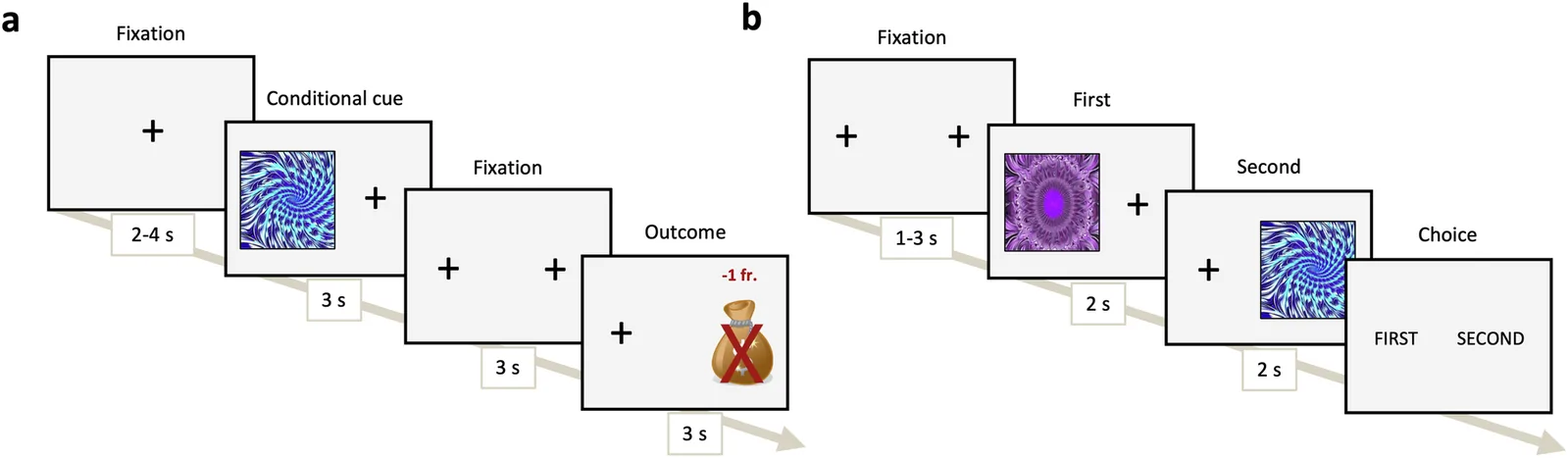
Schema of the Pavlovian conditioning task. **a** Pavlovian conditioning and (**b**) Forced choice task timeline.

The Pavlovian conditioning task consisted of 80 trials, in which five fractal images (Conditional Stimulus; CS) were associated with five different reward values (Unconditional Stimulus; US): two negative values of -2 CHF and -1 CHF, one neutral value of 0 CHF, and two positive values of +1 CHF and +2 CHF. The identity of the fractal (CS) predicted the reward value (US). At each trial, a fractal stimulus was presented on the right or left side (randomised across trials) of the screen for 3 s, followed by fixation crosses at the two potential stimulus locations during 3 s (Fig. 2). The reward value was then presented on the opposite side. Participants were instructed to observe the stimulus and memorise the fractal-value pairs.

We recorded the participant’s fixation time on the various locations (CS, US, and background) through the SMI RED 500 eye tracking system, recording binocularly at 500 Hz. The eye-gaze index was computed using the proportion of fixation time on the cue location minus the outcome location during the last second of the cue presentation.

We measured the sign- or goal-tracking bias by extracting the regression coefficient of a linear regression predicting the gaze index from the reward value of the CS based on the work of^37^. A higher learning regression coefficient signified a larger effect of the CS value and thus a higher sign-tracking bias (see Supplementary Material Section 2 for detailed computations).

##### Forced choice task

To assess whether the Pavlovian learning took place during the previous phase, participants then performed a forced choice task of 30 trials in which they had to choose the CS with the highest associated value between two sequentially presented CSs. They were instructed to choose the fractal with the highest value and that they would receive 10% of the reward value associated with the chosen CS. Stimuli were presented one at a time for 2 s, after which the participants selected the first or second stimulus. Each possible CSs pairing was presented 3 times in a randomised order. Participants were asked to respond faster if their response was above 2 s.

#### Sequential learning task

**Fig. 3:**
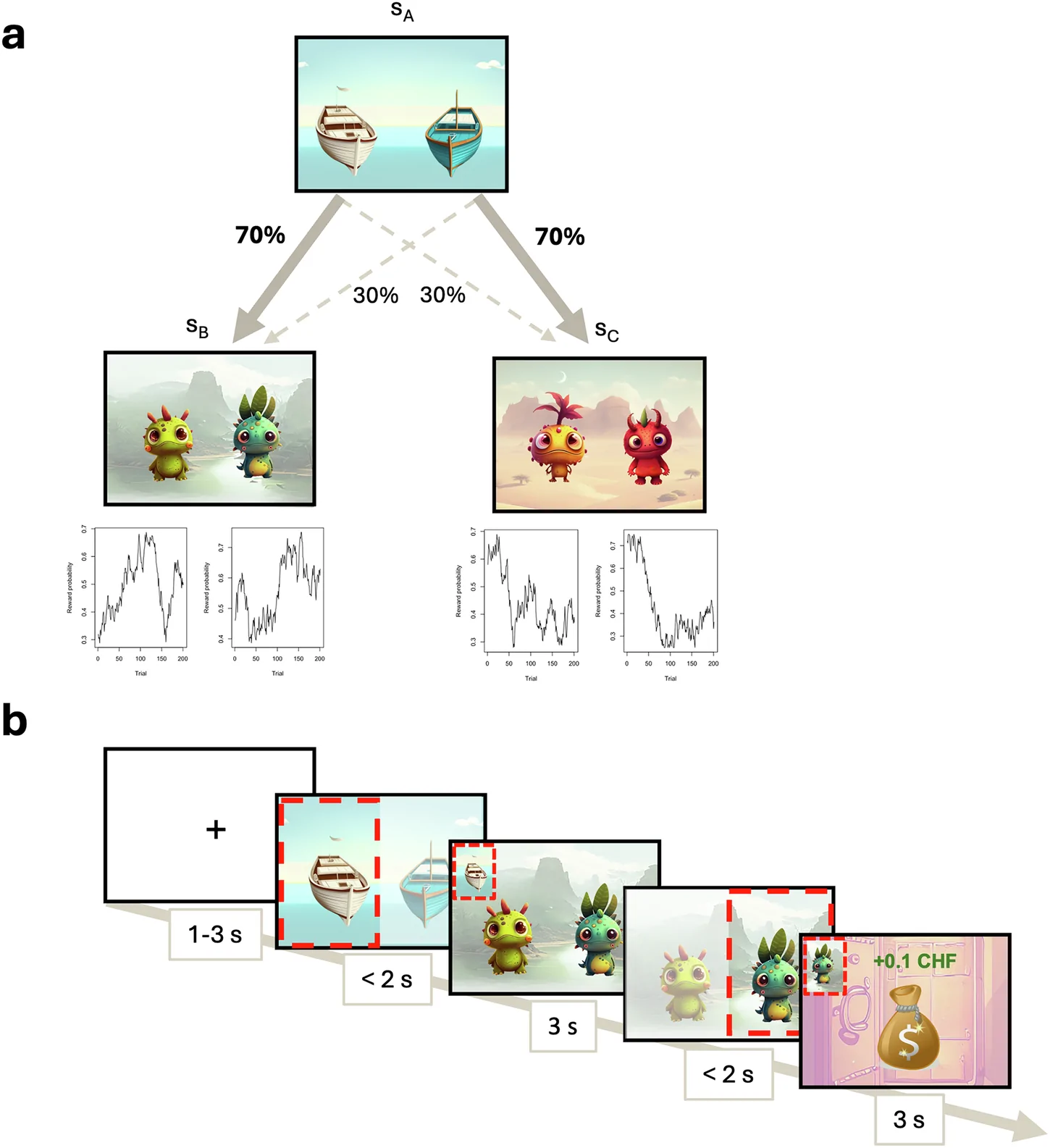
Schema of the Sequential Learning task. **a** The first choice between two boats at s_A_, each boat leads preferentially to one of the two islands s_B_ or s_C_. Each monster on the islands leads to a reward with a varying probability. **b** The Sequential Learning task timeline.

The method for the sequential learning task was replicated from our previous work^40^. The sequential learning task^19,38^ consisted of 201 trials, subdivided into blocks of 67 trials with breaks. Participants underwent extensive instructions about the task before starting the experiment. Each trial consisted of two subsequent stages with choices. At stage 1, participants chose between a white or blue boat, with each boat leading to one island of the two islands with a fixed probability (*P* = 0.7 or *P* = 0.3). At stage 2, participants chose between two monsters, with the choice of each monster being associated with a reward probability varying across trials. To encourage ongoing update of choice value, the probability of reward associated with each monster varied across the experiment following a random Gaussian walk (boundaries at 0.25 and 0.75, mean 0, SD 0.025; Fig. 3a)^38^. The trial started with a 1-3 s fixation cross, each choice was presented for 3 s and participants then had 2 s to make their choice at each stage.

The position of the boats and monsters was pseudorandomly (*p* = 0.5) permuted between the left and right of the screen across trials.

Reinforcement learning computational modelling was used to calculate the reinforcement learning bias during the first-stage action (i.e., boat selection). The pure model-free value was computed using a temporal difference (TD) algorithm, updating action values based solely on received rewards from previous trials, whereas the model-based value incorporated the boat-island transition probabilities in its estimation of action value. The value of the participant’s actual first-stage choice (${Q}_{{net}}$) is a weighted combination of the model-based and model-free computational models $({Q}_{{net}}=w{Q}_{{MB}}+(1-w){Q}_{{TD}})$. The weighting parameter w of this equation was used as the index of the learning bias. A low *w* indicating a stronger reliance on the model-free strategy (cf^19,38^., Supplementary Material Section 3 for detailed computations and parameter estimation).

#### Data quality checks

Specific data exclusion rules related to each outcome measure are described in the appropriate sections of the analysis plan (see Analysis plan for details). Floor effect will be examined for the consumption variable, and defined as >15% of observations at the minimum score^41,42^. If such an effect is detected, the corresponding hypothesis (H2) would not be interpreted. Additionally, for all outcomes, participants outside a 3× Median Absolute Deviation (MAD) range around the median (conservative value for outlier exclusion^43^) would be excluded from the respective analysis (see Analysis plan for details).

### Sampling plan

For all analyses, we determined the smallest effect size of interest (SESOI) as small to medium (i.e., coefficient of 0.4 for correlations; R^2^ of 0.16 for linear regression). The chosen threshold aligns with effect sizes reported in similar studies^4,19,35,37^ and represents a lower bound that can be reliably found in the literature while still large enough to be relevant. This threshold ensures theoretical and practical relevance while preventing the overinterpretation of trivial effects, following established methodological principles^44,45^.

The power analysis script can be downloaded on our OSF study page (see Code Availability Statement).

#### Learning biases are correlated with self-reported cravings for HCHP foods

For both Pavlovian bias (H1a) and reinforcement learning bias (H1b), we determine an SESOI to be a correlation of 0.4 (small to medium effect) for the association between learning biases and self-reported cravings. The a priori power analysis conducted with the pwr R package^46^ with an alpha of .05 indicates that 63 participants are required to reach a power of .95 for the target one-sided correlation.

#### Learning biases moderate the relationship between self-reported cravings and consumption of HCHP foods

For both Pavlovian bias (H2a) and reinforcement learning bias (H2b), we determine an SESOI for the impact of the interaction between self-reported cravings of HCHP food and learning bias on HCHP food consumption to be at least an R^2^ of 0.16. A priori power analysis conducted with the pwrss R package^47^ indicates that 76 participants are necessary to reach a power of .95 with an alpha of .05 for a linear model with a continuous outcome (consumption of HCHP food), a continuous moderator (Pavlovian learning bias, reinforcement learning bias), and a continuous predictor (self-reported cravings of HCHP food).

#### Learning biases are correlated with approach bias for HCHP foods

For both Pavlovian bias (H3a) and reinforcement learning bias (H3b), we determine an SESOI to be a correlation of 0.4 (small to medium effect) for the association between learning biases and approach bias. The a priori power analysis conducted with the pwr R package^46^ with an alpha of .05 indicates that 63 participants are required to reach a power of .95 for the target one-sided correlation.

Due to a floor effect observed for H2 during recruitment, we conducted a floor effect analysis to assess whether further recruitment would mitigate this issue. This indicated that the floor effect would persist despite increasing the sample size (see Section “H2) Learning biases moderate the relationship between self-reported cravings and consumption of HCHP foods”). The final sample target was thus revised to match that of H1 and H3. Recruitment continued until the post-exclusion sample included 63 participants with complete datasets. Distribution outliers were not planned to be replaced to avoid circularly changing the thresholds for exclusion.

### Analysis plan

#### Preprocessing procedures

##### Daily self-reported craving

Participants completing less than 50% of the daily self-reported craving questionnaires were excluded from the analysis and replaced if this meant dropping below the planned sample size.

The self-reported craving was measured every day and computed as the number of HCHP food cravings multiplied by the intensity of the cravings (ranging from 0 to 1), divided by the total number of daily assessments:

$${Daily}\,{craving}=\frac{\sum {HCHP}\,{cravings}\,{occurence}\times {intensity}\,}{\sum {daily}\,{assessments}}$$

Participants with no reported HCHP cravings across all daily assessments were assigned a mean daily craving of 0 and a standard deviation of 0.

Participants outside a 3×MAD range around the median of the participants’ average daily craving (H1a first model, H1b first model, H2) and around the median of participants’ standard deviation of daily cravings (H1a second model, H1b second model) were excluded from the respective analyses.

##### Daily self-reported consumption

Participants completing less than 50% of the daily self-reported consumption questionnaires were excluded from the analysis and replaced if this meant dropping below the planned sample size.

The self-reported consumption was measured every day and computed as the number of HCHP food consumption events, defined as eating events within a category that exceed a minimum of 200 kcal - a threshold reflecting a typical snacking event for HCHP foods (e.g., 1 croissant ≈ 230 kcal, 1 small ice cream cup ≈ 200 kcal).

$${Daily}\,{consumption}=\frac{\sum {HCHP}\,{consumption}\,{events}\,}{\sum {daily}\,{assessments}}$$

If a floor effect at the population level is detected (see Data quality checks for details), the hypothesis would not be interpreted.

Participants outside a 3×MAD range around the median of the participants’ average daily consumption were excluded from the respective analysis (H2).

##### Approach biases

We excluded trials with reaction times below 250 ms and above 2.5 s. We only considered trials with correct responses.

The approach bias was measured every other day and was computed as a double-difference score as in^36^, by subtracting the difference between the session averaged reaction time for approach of HCHP and objects from the difference between the session averaged reaction time for avoidance of HCHP and objects.

$${Approach}\,{bias}=([{HCHP}\,{avoidance}]\,-\,[{HCHP}\,{approach}])\,-\,(\left[{Object}\,{avoidance}\right]-\,\left[{Object}\,{approach}\right])$$

Participants outside a 3×MAD range around the median of the participants’ average approach bias (H3a first model, H3b first model) and around the median of participants’ standard deviation of approach bias (H3a second model, H3b second model) were excluded from the respective analysis.

##### Pavlovian bias

Participants with an error rate above 10% in the Forced Choice task were excluded from the analysis and replaced, if this meant dropping below the planned sample size.

The Pavlovian bias was computed as the regression coefficient of a linear regression predicting the gaze index from the reward value of the CS. A higher learning regression coefficient signifies a larger effect of the CS value and, thus a higher sign-tracking bias.

Participants outside a 3×MAD range around the median for the Pavlovian bias were excluded from the respective analyses (H1a, H2a, H3a).

##### Reinforcement learning bias

We excluded trials with reaction times below 250 ms or omitted response (above 2 s).

Participants with more than 20% of excluded trials, as well as participants with a probability of repeating the first-stage action following a common rewarded trial worse than chance, were excluded from the respective analyses (H1b, H2b, H3b) and replaced if this meant dropping below the planned sample size.

The reinforcement learning bias was computed as the weighting parameter *w* for the combination of the model-based and model-free computational models in the value of the participant’s first choice’s action. A low *w* indicated a stronger reliance on the model-free strategy.

Participants outside a 3×MAD range around the median for the reinforcement learning bias were excluded from the respective analyses (H1b, H2b, H3b).

#### Learning biases are correlated with self-reported cravings for HCHP foods

We computed the correlation between the cravings for HCHP foods and the learning biases (H1a: Pavlovian bias, H1b: reinforcement learning bias). To capture both inter- and intra-individual variability, the cravings for HCHP foods were defined as i) the mean of daily cravings and ii) the standard deviation of daily cravings over the two-week study period. Using both measures enables a comprehensive assessment of craving dynamics: while the mean reflects the general tendency or average intensity of cravings, the standard deviation captures the magnitude of fluctuations in craving intensity over time. As such, two correlations would be computed for both H1a and H1b. A Bonferroni correction was applied to account for multiple testing (alpha of 0.025). We hypothesised that Pavlovian bias would be positively correlated with both the mean and the standard deviation of self-reported cravings for HCHP foods (H1a). While still wanting to investigate a potential relationship between reinforcement learning bias and cravings for HCHP foods (H1b), we are less confident that it exists (see Introduction). We hypothesised that the model-free bias (lower w) would be positively correlated with the mean and negatively correlated with the standard deviation of self-reported cravings for HCHP foods (H1b).

#### Learning biases moderate the relationship between self-reported cravings and consumption of high-caloric highly palatable (HCHP) foods

We computed the correlation between the cravings for HCHP foods and the learning biases. We looked at the daily self-reported cravings x learning bias (H2a: Pavlovian bias, H2b: reinforcement learning bias) interaction term of a mixed linear model with the participant as a random within-effect and the daily self-reported consumption for HCHP foods as the outcome (i.e., consumption ~ cravings * learning bias + (cravings|participant)). We expected an effect of the interaction term between Pavlovian/reinforcement learning bias and cravings for HCHP foods on HCHP foods consumption.

These models would be computed using the lmer() function from the lmerTest package in R^48^.

#### Learning biases are correlated with approach bias for HCHP foods

We computed the correlation between the approach bias for HCHP foods and the learning biases (H3a: Pavlovian bias, H3b: reinforcement learning bias). To capture both inter- and intra-individual variability, the approach bias for HCHP foods was defined as i) the mean of approach biases and ii) the standard deviation of approach biases over the two-week study period. Using both measures enabled a comprehensive assessment of the approach bias dynamics: while the mean reflected the general approach bias tendency, the standard deviation captured the magnitude of fluctuations over time. As such, two correlations were computed for both H3a and H3b. A Bonferroni correction was applied to account for multiple testing (alpha of 0.025). We hypothesised that Pavlovian bias would be positively correlated with both the mean and standard deviation of the approach bias for HCHP foods (H3a). While still wanting to investigate a potential relationship between reinforcement learning bias and approach bias for HCHP foods (H3b), we are less confident that it exists (see Introduction). We hypothesised that the model-free bias (lower w) would be positively correlated with the mean and negatively correlated with the standard deviation of approach bias for HCHP foods (H3b).

#### Preregistered exploratory analysis

Given that sign-tracking biases and approach bias both capture automatic processes, they may share a more direct connection with approach bias compared to self-reported cravings. However, although approach bias may reflect one automatic component of craving, namely a higher tendency to approach food-related stimuli, the learning biases may be associated with other components of cravings (e.g., habit, influence of environmental and physiological cues), leading to stronger associations with the overall subjective experience of craving than with approach bias alone. Moreover, while implicit measures are theoretically designed to capture automatic processes, self-reported measures may better reflect the subjective reality of participants^49^ and align with participants’ lived experiences or preferences. Therefore, we included an exploratory analysis to examine whether there is a stronger association between learning biases and either approach bias or self-reported cravings.

We compared correlations between self-reported cravings and approach biases for both learning biases. To capture both inter- and intra-individual variability, we used two measures: (i) the mean daily cravings or approach biases and (ii) the standard deviation of daily cravings or approach biases over the two-week study period. We expected a difference of r of approximately 0.2 between the self-reported and approach bias correlations for at least one outcome (i.e., mean or standard deviation), which would be sufficient to shift the effect size classification (e.g., from small to moderate). A higher correlation in approach bias would suggest learning biases are more directly linked to the captured automatic processes, whereas a higher correlation in self-reported cravings would indicate that learning biases are more closely linked to subjective experience rather than automatic processes. Differences in correlation for the mean would suggest one measure captures craving intensity better, while differences in the standard deviation would point to differences in capturing craving variability. If no difference of at least 0.2 is observed, this would imply that both measures capture similar aspects of the relationship or that the link between cravings and learning biases is too weak to distinguish between them. Full analysis with results can be found in the Supplementary Material (Section 7.3; Supplementary Fig. 3).

Additionally, we reported the Bayes factor (BF_01_) for all confirmatory hypotheses to provide additional insight into the strength of evidence for or against the null hypothesis. For H1 a value above 10 will indicate additional evidence supporting the absence of a relationship between the relevant learning bias and cravings. For H2, a value above 10 will provide evidence supporting the absence of an interaction between cravings and the relevant learning bias in predicting consumption. For H3, a value above 10 will indicate evidence supporting the absence of a relationship between the relevant learning bias and approach bias.

## Results

In total, 81 participants were eligible and completed the two in-lab assessments (see Supplementary Fig. 1 for sample size progression). Participants had a mean age of 25.8 years (SD = 7.6) and a mean body mass index (BMI) of 22.3 kg/m² (SD = 2.1). The sample comprised 55 women and 26 men (proportion female = 0.68). The mean questionnaire completion rate was 73.1% (SD = 23.2), and the mean mobile approach-avoidance task (mobile-AAT) completion rate was 69.3% (SD = 28.8).

All results were reported alongside Bayes factors as complementary information. However, these were not used as a basis for interpretation.

### Learning biases are correlated with self-reported cravings for HCHP foods

#### Learning bias as Pavlovian bias

For H1a, nine participants were excluded due to low Ecological Momentary Assessments (EMA) response rates ( < 50%), one due to a technical issue in the Pavlovian task, and four due to high error rates on forced-choice trials in the Pavlovian task. In total, eleven participants were excluded, resulting in a sample of 70 prior to outlier removal. Ten outliers were subsequently removed for the Pavlovian bias. There were no outliers for the mean of high-caloric highly palatable (HCHP) daily cravings, resulting in 59 participants included in the first model. There was one outlier for the standard deviation of HCHP daily cravings, resulting in 58 participants included in the second model.

**Fig. 4:**
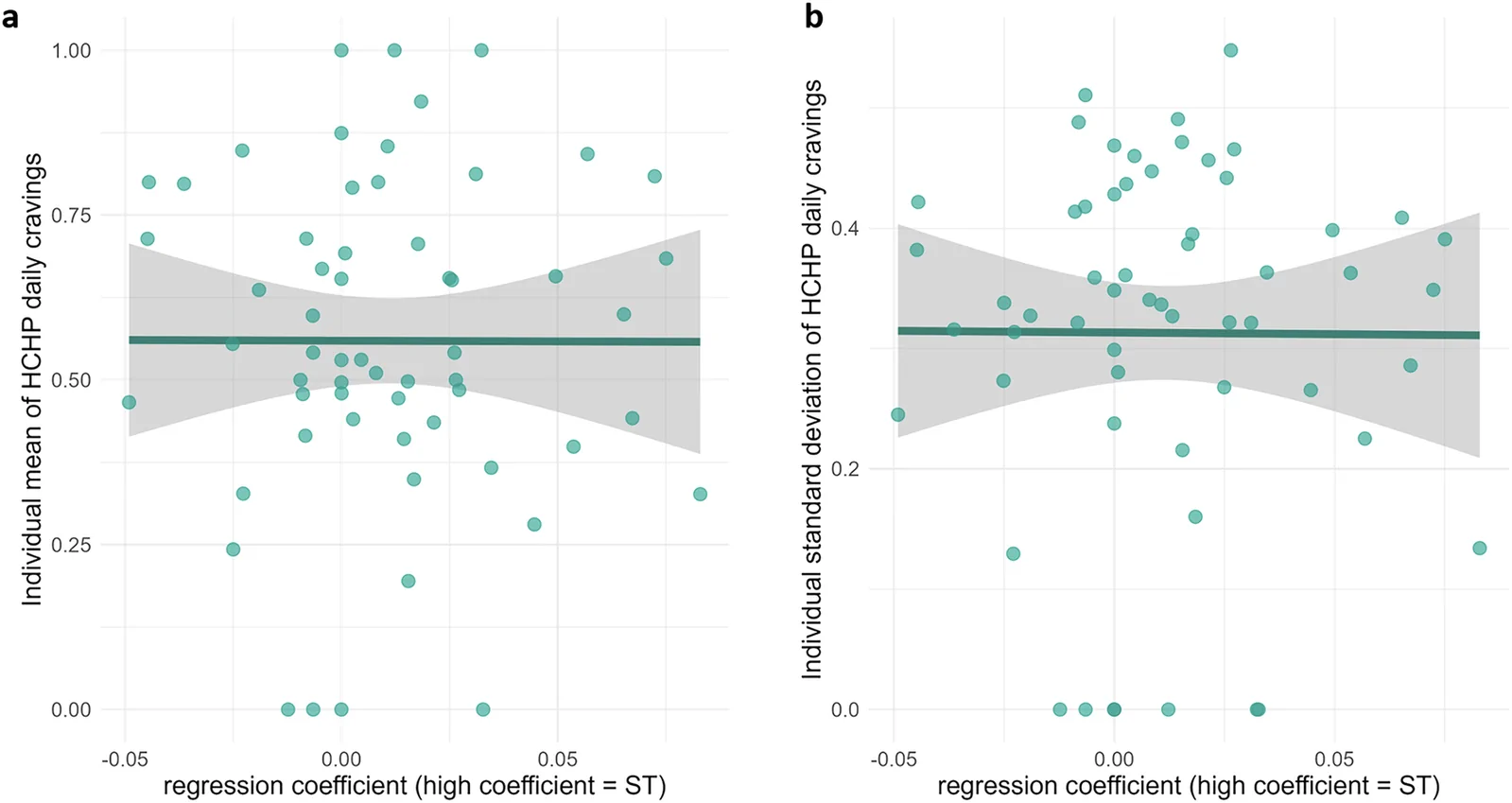
Scatterplots of the Pavlovian regression coefficient and HCHP cravings. Cravings are expressed as **a** the mean (*n* = 59) and **b** the standard deviation of daily cravings (*n* = 58). Individual data points (coloured dots), the fitted regression line (coloured line), and the 95% confidence interval (grey shaded area) are shown.

We did not observe a positive relationship between the sign-tracking bias and mean cravings (r = -0.003, 95% CI = [-0.219, 1.000], t(57) = -0.019, p = .508; Fig. 4a). The Bayes factor provided moderate evidence in favour of the null hypothesis (BF_01_ = 3.78).

We also did not observe a positive relationship between the sign-tracking bias and craving variability (r = -0.006, 95% CI = [-0.224, 1.000], t(56) = -0.042, p = .517; Fig. 4b). The Bayes factor provided moderate evidence in favour of the null hypothesis (BF_01_ = 3.76).

#### Learning bias as reinforcement learning bias

For H1b, EMA questionnaire exclusions were the same as above (nine participants excluded). Seven participants were excluded from the *w* bias analysis due to violations of task-specific criteria. In total, 16 participants were excluded or had missing data, leaving a final sample of 65 before outlier removal. No outliers were identified for the *w* bias. Three outliers were identified for the mean of HCHP cravings, leaving a total of 62 participants for the first model. Eight outliers were identified for the standard deviation of cravings, leaving a total of 57 participants for the second model.

**Fig. 5:**
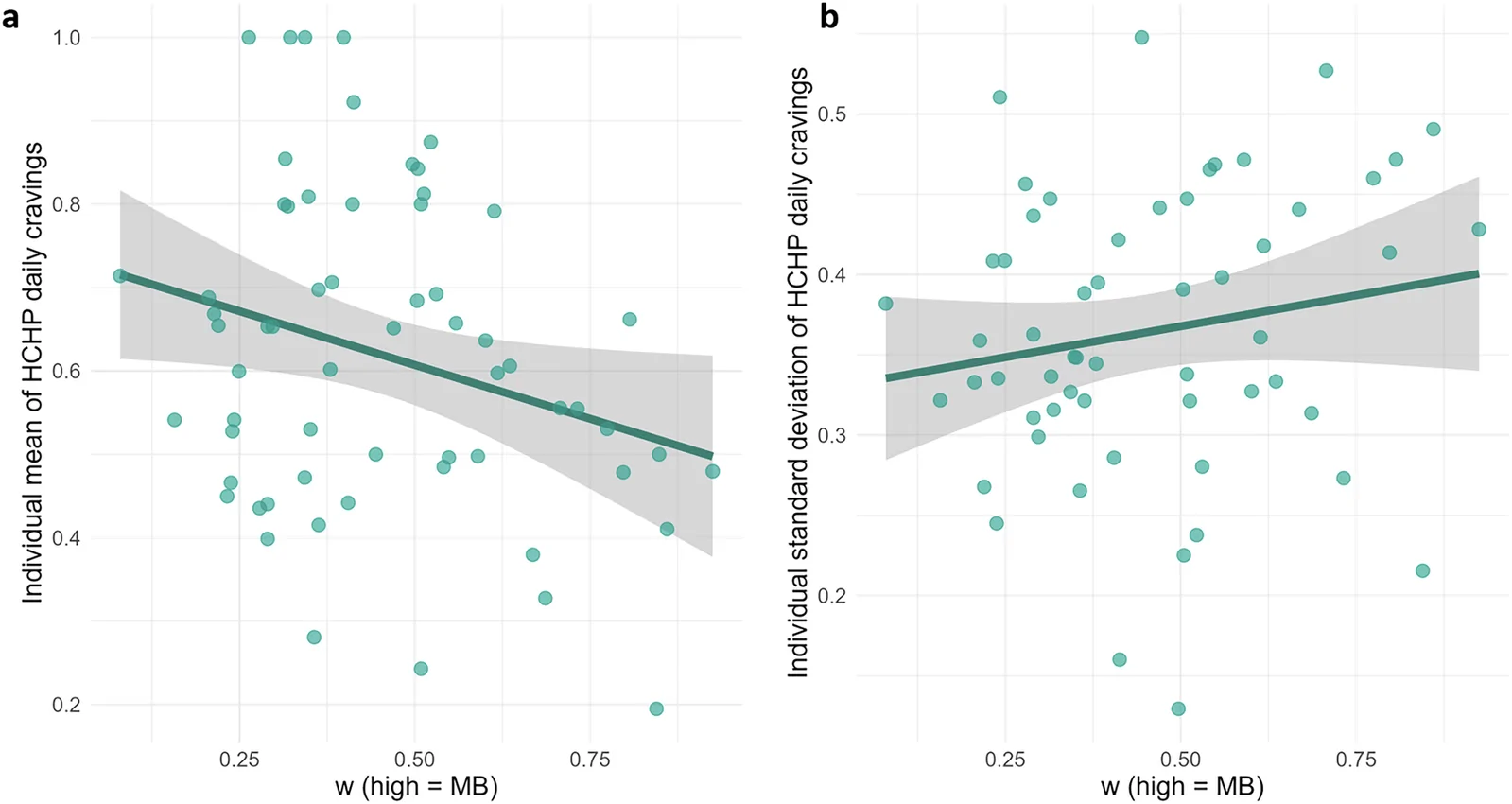
Scatterplots of the w index and HCHP cravings. Cravings are expressed as **a** the mean (*n* = 62) and **b** the standard deviation of daily cravings (*n* = 57). Individual data points (coloured dots), the fitted regression line (coloured line), and the 95% confidence interval (grey shaded area) are shown.

We observed a significant medium negative association between *w* (lower *w* = higher model-free bias) and the mean of daily HCHP cravings (r = -0.270, 95% CI = [-1.000, -0.062], t(60) = -2.171, *p* = .018; Fig. 5a), which survived our corrected significance threshold (*p* < .025). The Bayes factor provided anecdotal evidence in favour of the null hypothesis (BF_01_ = 1.03).

We did not observe a positive association between *w* and craving variability (r = 0.172, 95% CI = [-0.050, 1.000], t(55) = 1.293, p = .101; Fig. 5b). The Bayes factor provided weak evidence in favour of the null hypothesis (BF_01_ = 1.86).

### Learning biases moderate the relationship between self-reported cravings and consumption of HCHP foods

Nine participants were excluded due to low consumption answer rate, resulting in 72 participants. A floor effect was observed on that sample, with more than 15% of daily consumption assessments at the population level (18.24%).

An analysis was thus conducted to assess whether increasing the sample size could mitigate the observed floor effect.

Out of 81 participants, 72 met the inclusion criterion of a consumption answer rate greater than 0.5. Based on the most conservative scenario observed in H1b, 9% of participants were subsequently excluded due to bias-related criteria (65 retained out of the 72). To obtain a final sample of 76 participants after such exclusions, approximately 85 participants would be required prior to exclusion (76 / 0.909 ≈ 84.4). Given that 68 participants currently had usable consumption data, this corresponds to an estimated need for 16 additional participants.

To evaluate whether further recruitment would meaningfully reduce the floor effect, a bootstrap simulation was performed. Specifically, additional participants were repeatedly sampled from the empirical distribution of the existing data (10,000 iterations), and the projected proportion of zero consumption values was recomputed.

**Fig. 6:**
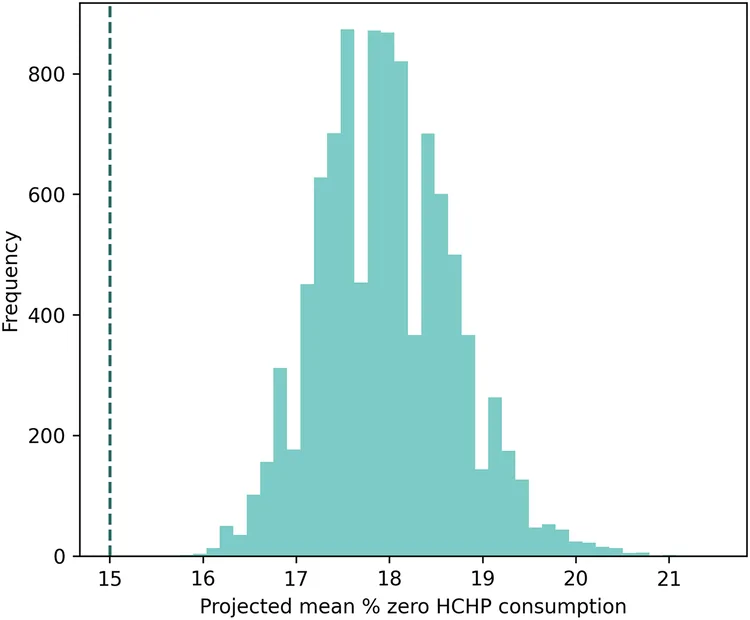
Projected distribution of the mean percentage of zero HCHP consumption. The histogram shows the distribution obtained from 10,000 bootstrap simulations after adding 16 simulated participants (*n* = 85) to the empirical sample. The dashed vertical line indicates the predefined floor-effect threshold (15%). All simulated outcomes remain above this threshold, indicating that the floor effect would persist even with additional recruitment.

The projected mean percentage of zero consumption remained stable (Fig. 6; mean = 17.96%, 95% CI = [16.640, 19.481]). Critically, the probability that the floor effect would persist (defined as ≥15% zero values) was 1.0, while the probability of observing no floor effect was 0.0.

These results indicate that increasing the sample size would not have mitigated the floor effect. Continuing recruitment under these conditions would have been neither scientifically justified nor ethically appropriate, thus justifying our decision to stop recruitment.

As preregistered, the hypothesis was not tested, and the results are not reported here but can be found in the Supplementary Material (Section 7.1 and 7.2).

### Learning biases are correlated with approach bias for HCHP foods

#### Learning bias as Pavlovian bias

For H3a, four participants were excluded due to high error rates in the forced-choice task. Six participants had no approach bias data, resulting in 72 participants included in the analysis. Two participants had only one daily approach bias measure and thus no data for the standard deviation of daily approach bias, resulting in 70 participants for the second model of this analysis. Seven participants were excluded due to Pavlovian outliers. Two participants were excluded due to mean approach bias outliers, resulting in a sample of 62 participants for the first model. Two participants were excluded due to standard deviation approach bias outliers, resulting in a sample of 60 participants for the second model.

**Fig. 7:**
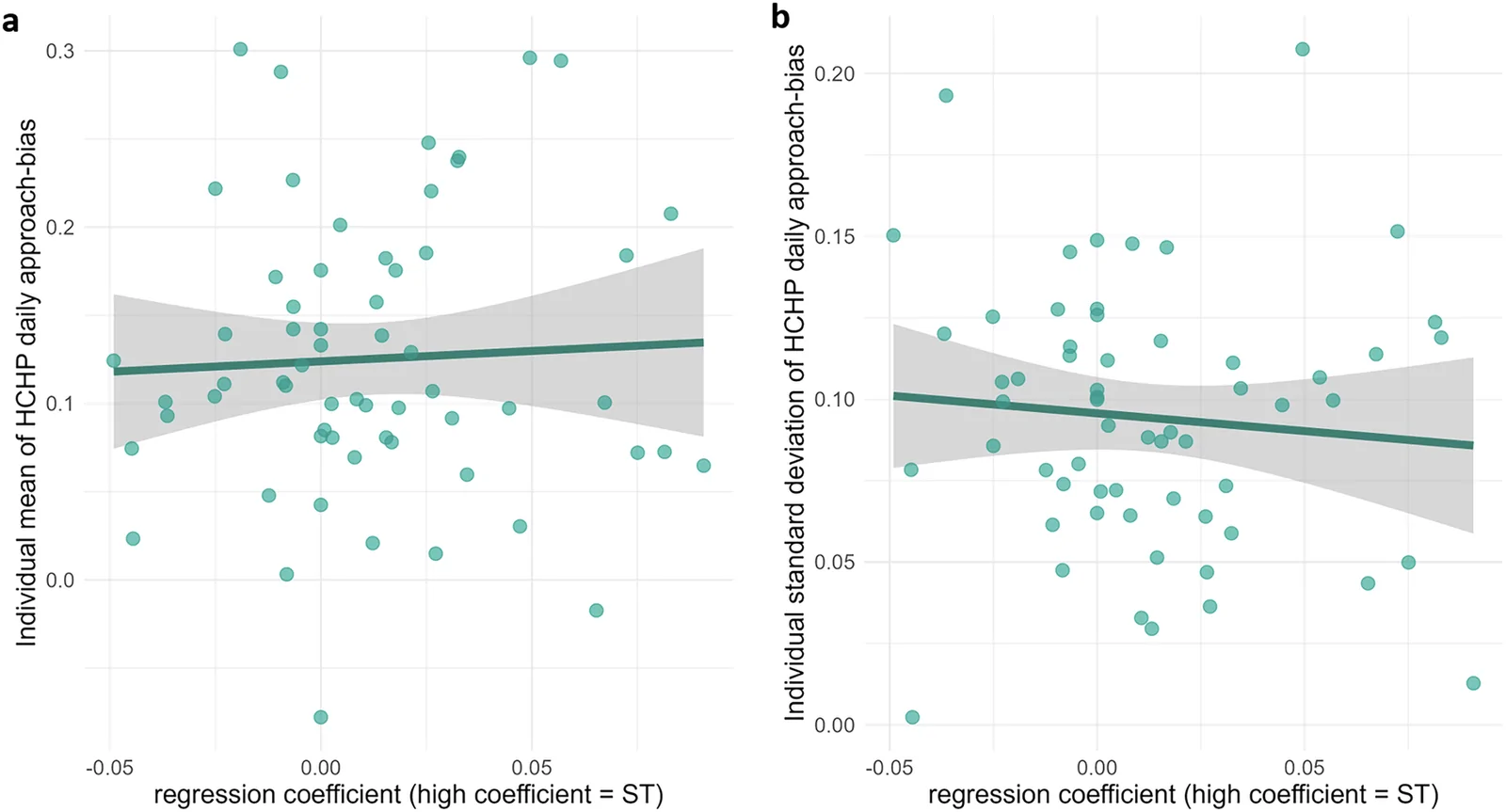
Scatterplots of the Pavlovian regression coefficient and HCHP approach bias. Approach bias is expressed as **a** the mean (*n* = 62) and **b** the standard deviation of daily approach biases (*n* = 60). Individual data points (coloured dots), the fitted regression line (coloured line), and the 95% confidence interval (grey shaded area) are shown.

We did not observe a positive relationship between the sign-tracking bias and approach bias (r = 0.048, 95% CI = [-0.164, 1.000], t(60) = 0.373, p = .355; Fig. 7a). The Bayes factor provided moderate evidence in favour of the null hypothesis (BF_01_ = 3.64).

We did not observe a positive relationship between the sign-tracking bias and approach bias variability (r = -0.090, 95% CI = [-0.298, 1.000], t(58) = -0.686, p = .752; Fig. 7b). The Bayes factor provided moderate evidence in favour of the null hypothesis (BF_01_ = 4.01).

#### Learning biases as reinforcement learning bias

Seven participants were excluded for violating the sequential learning task criteria, and six additional participants had no approach bias data, resulting in 68 participants. One further participant was excluded as an outlier in the mean approach bias, resulting in a sample of 67 participants in the first model of this analysis. One participant had no standard deviation data and two participants had outliers for the standard deviation of approach bias, resulting in 64 participants included in the second model of this analysis.

**Fig. 8:**
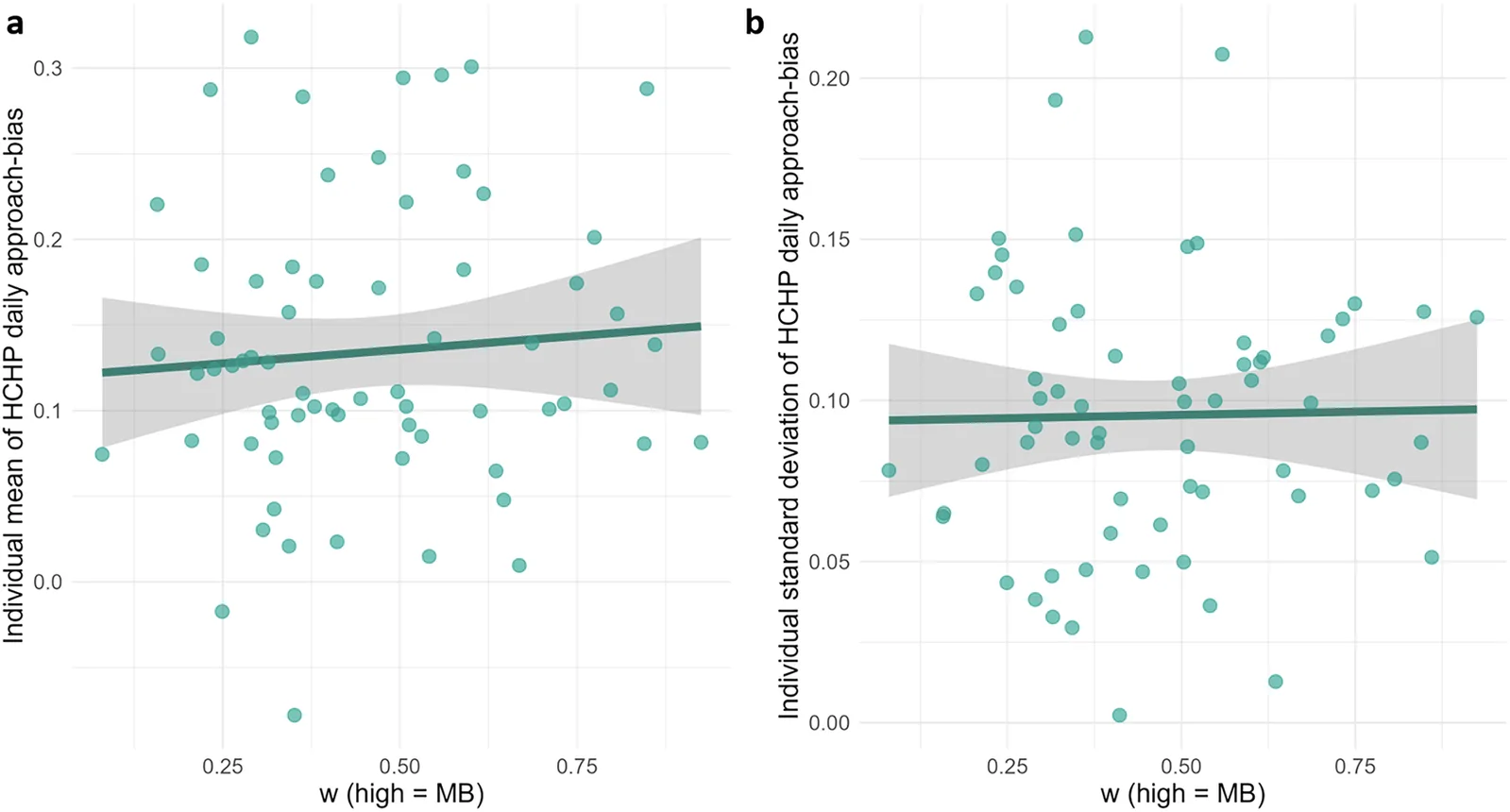
Scatterplots of the bias w and HCHP approach bias. Approach bias is expressed as **a** the mean (*n* = 67) and **b** the standard deviation of daily approach biases (*n* = 64). Individual data points (coloured dots), the fitted regression line (coloured line), and the 95% confidence interval (grey shaded area) are shown.

We did not observe a significant positive relationship between *w* and the mean approach bias (r = 0.078, 95% CI = [-1.00, 0.28], t(65) = 0.624, p = .733; Fig. 8a). The Bayes factor provided weak to moderate evidence in favour of the null hypothesis (BF_01_ = 3.38).

We did not observe a significant negative relationship between *w* and the standard deviation of approach bias (r = 0.019, 95% CI = [-1.000, 0.225], t(62) = 0.146, p = .558; Fig. 8b). The Bayes factor provided moderate evidence in favour of the null hypothesis (BF_01_ = 3.88).

## Discussion

We conducted a two-week longitudinal study to examine whether sign-tracking and model-free learning biases were associated with high-caloric, highly palatable (HCHP) food craving and intake in everyday life. Using ecological momentary assessments delivered via a smartphone app, we measured daily craving, food consumption, and approach-avoidance tendencies in real-world contexts. Learning biases were indexed using Pavlovian conditioning and sequential learning tasks. We hypothesised that stronger Pavlovian or model-free biases would be associated with increased cravings (H1), moderate the craving-consumption relationship (H2), and be associated with stronger approach bias (H3) for HCHP foods.

### Learning bias and self-reported eating behaviours

The first hypothesis examined whether learning biases were associated with self-reported cravings for HCHP foods. The sign-tracking bias was not associated with cravings, but the model-free learning bias was, as driven by greater average HCHP daily cravings for model-free individuals (i.e., lower *w* index).

In contrast to our hypothesis, sign-tracking bias was not associated with daily HCHP cravings, neither in their average intensity nor in their variability. Importantly, the Bayes factors (BF_01_ = 3.78 and 3.76, respectively) provided moderate evidence in favour of the null hypothesis, indicating that the absence of association is unlikely to reflect insufficient sensitivity and constitutes a substantive finding under our preregistered analysis plan. This tempers the assumption that sign-tracking, as indexed by the eye-gaze regression coefficient on the Pavlovian conditioning task, generalises to aggregate craving intensity in everyday life among healthy-weight adults. It does not, however, preclude associations with more circumscribed outcomes. Sign-tracking has been linked to cue valuation^10^, adiposity, and altered neural responses to food cues^50^, suggesting that its relevance for eating behaviour may lie in cue-induced rather than aggregate cravings, in cue-reactivity paradigms, or in populations with stronger HCHP intake patterns.

We found that model-free bias was positively correlated with self-reported cravings for HCHP foods, as expressed through a positive association of the model-free bias and the average of self-reported HCHP cravings. This association with model-free learning might reflect a reinforcing feedback loop. For instance, habitual model-free behaviours, such as consistently consuming chocolate after lunch, could amplify anticipatory physiological reactions (e.g., salivation, gastric activity) consciously perceived as cravings. Additionally, model-free learning updates action values based on reward prediction errors, continuously reinforcing the perceived value of craved items. Taken together, these findings provide preliminary evidence that action-related reinforcement learning tendencies are implicated in everyday eating behaviour, although further research is required to clarify the nature and extent of this implication. Future work could, for example, assess whether the observed effects are specific to HCHP cravings or generalise to cravings for other food categories, including healthier options. In addition, subsequent studies could focus on outcomes indexing habitual eating patterns rather than aggregate craving or consumption measures, such as timing, food type, and portion size.

Importantly, the present study investigated HCHP cravings and consumption in a healthy-weight sample, which may exhibit limited HCHP cravings and consumption patterns, thereby preventing adequate characterisation of their underlying mechanisms. For example, food cues other than HCHP may still acquire incentive salience in sign-trackers. Similarly, model-free habitual eating behaviours may be observed outside of HCHP foods. Future work could thus benefit from targeting populations characterised by higher levels of HCHP cravings and intake, or observing broader food categories.

The second hypothesis investigated the moderating effect of the learning biases on the relationship between daily cravings and consumption. Due to an observed floor effect, this hypothesis could not be tested as per preregistration.

### Approach bias and learning biases

The final hypothesis tested whether learning biases were associated with HCHP approach bias measured with the mobile approach-avoidance task (mobile-AAT). Neither bias was significantly related to the approach bias index. However, it remains difficult to determine whether this absence of association reflects a genuine lack of relationship or limitations of the approach bias measure itself. There are indeed concerns in the literature regarding the reliability and state-dependency of approach-avoidance measures^50,51^, and these tasks may be better suited to study within-person fluctuations rather than between-person differences, as in the present design. Moreover, the considerable variability observed in answer rates for the mobile-AAT also calls into question the measure’s effective statistical power, potentially limiting the reliability of its estimates.

### Limitations

Several limitations should be considered when interpreting these findings. First, the learning bias indices were assessed at single in-lab sessions, and the daily-life outcomes were observational. Causal claims about how habitual model-free processes shape craving dynamics, or conversely how repeated craving episodes reinforce model-free tendencies, therefore go beyond what the present data can support. Second, the heterogeneity in answer rates for the Mobile-AAT (69.3 ± 28.8%) introduces variability in the reliability of individual estimates for between-person comparisons of the approach bias index, whereas this limitation was mitigated in the Ecological Momentary Assessments (EMA) questionnaire with exclusions for participants with low answer rates. Third, the two-week sampling window may be insufficient to capture longer-cycle craving dynamics or context-dependent fluctuations. Fourth, the sample was restricted to healthy-weight adults, who may exhibit limited HCHP craving and consumption patterns and thereby constrain the range of behaviour available to detect cue-driven effects, particularly for sign-tracking. Replication in populations with elevated HCHP intake, alongside repeated assessment of the learning-bias indices and within-person designs better suited to approach-avoidance measures, would strengthen the inferences that can be drawn about the role of these biases in everyday eating.

## Conclusion

This study investigated whether Pavlovian and model-free learning biases were associated with high-caloric highly palatable (HCHP) food craving and intake in everyday life, using a two-week longitudinal design with ecological momentary assessments delivered via a smartphone app. Results indicated that sign-tracking bias was not meaningfully associated with HCHP craving, whereas the model-free learning bias was significantly associated with HCHP cravings as expressed through higher mean cravings. Given well-established evidence linking sign-tracking to cue valuation, these null findings might not indicate an absence of influence on eating behaviour as a whole but rather call for further research to clarify the role of Pavlovian learning biases within this framework. In contrast, the positive association between model-free bias and craving provides preliminary evidence for the role of habit-like reinforcement processes in everyday craving experiences. Approach bias was not associated with either learning bias, which might be due to task limitations and answer rate variability, and highlights an important limitation of between-person approach bias designs. Overall, the present work suggests that reinforcement learning tendencies may contribute to everyday eating behaviour, and future work could examine more diverse samples, broader food categories, and more direct indices of habitual eating and cue-induced responding.

## Supplementary information

Transparent Peer Review file

Supplementary Material

## Data Availability

The data for Stage 1 and Stage 2 are available on the study OSF page (https://doi.org/10.17605/OSF.IO/43FYA)^52^. The Stage 1 protocol for this Registered Report was accepted in principle on June 20th, 2025. The protocol, as accepted by the journal, can be found at https://doi.org/10.6084/m9.figshare.29445518.

## Code availability

All scripts, including custom code for the sequential learning task, for Stage 1 and Stage 2 are available on the study OSF page (https://doi.org/10.17605/OSF.IO/43FYA)^52^.
