# Supplementary material for: The role of affective learning biases in daily food behaviour
Source: Commun Psychol. 2026 Jul 24;4:136. doi: 10.1038/s44271-026-00511-x (PMC13652808; doi:10.1038/s44271-026-00511-x)
Supplement: Supplementary file 1 [file 44271_2026_511_MOESM1_ESM.pdf]

nature portfolio

Peer Review File

The role of affective learning biases in daily food behaviour

Corresponding Author: Ms Malika Tapparel

This file contains all editorial decision letters in order by version, followed by all author rebuttals in order by version.

Version 0:

Decision Letter:

Dear Ms Tapparel,

Thank you once again for your manuscript, entitled "From craving to consumption: the role of affective learning biases in daily food behaviour," and for your patience during the peer review process.

Your manuscript has now been evaluated by 4 reviewers, with reviewer #3 and #4 co-reviewed with reviewer #1, whose comments are included at the end of this letter. Although the reviewers find your protocol to be of interest, they also raise some important concerns. We are very interested in the possibility of proceeding further with your submission in Communications Psychology, but would like to consider your response to these concerns in the form of a revised manuscript before we make a decision on in principle acceptance and Stage 2 submission.

To guide the scope of the revisions, the editors discuss the referee reports in detail within the team, including with the chief editor, with a view to (1) identifying key priorities that should be addressed in revision and (2) overruling referee requests that are deemed beyond the scope of the current study. We hope that you will find the prioritised set of referee points to be useful when revising your study. Please do not hesitate to get in touch if you would like to discuss these issues further.

The reviewers voice a number of key concerns, which pertain to 1) the strength of evidence the study can obtain given concerns about effect sizes and sensitivity, 2) the clarity and presentation of the hypotheses throughout, and 3) the presentation of the aims of the study. To address these concerns, we consider it necessary that the resubmitted protocol includes the following revisions:

In revision, you must include power-analyses for all three hypotheses. We will only be able to take forward a Stage 1 protocol that achieves 95% power for the smallest effect size of interest for each respective hypothesis. We note that you formulate a target effect size of .2 for hypothesis 3 in the Design Table, but the full power analysis is missing. Likewise missing are justifications for the respective effect size is the smallest effect size of theoretical or practical interest. Given the reviewers' concerns about the robustness of the analysis, we ask that a revision includes not only a strong justification of the respective effect size (ideally derived from multiple studies, not based on single pilot studies) and simulations that demonstrate that the (revised) analyses are feasible and informative in the current task design and analysis pipeline. To strengthen the revision, the protocol should include pilot data to demonstrate feasibility and interpretability of the human data that originate from this specific protocol. Please note that you will not be able to interpret null results unless you preregister a Bayesian analysis or an equivalence test.

Registered Reports must be confirmatory, which means that the hypotheses need to be clear and testable and presented in precise terms throughout. This applies not only to the Design Table and Methods, but should be adopted for the Introduction as well. The interpretation of alternative outcomes (i.e. a null finding or negative finding) needs to be noted at the least in the Design Table.

Where you present the key aims of the research, please clarify the theoretical contribution your study will make, avoiding claims about applied insights where these are not inherent in the design.

In sum, we invite you to revise your Stage 1 Registered Report taking into account reviewer and editor comments. Please highlight all changes in the manuscript text file.

We are committed to providing a fair and constructive peer-review process. Do not hesitate to contact us if there are specific requests from the reviewers that you believe are technically impossible or unlikely to yield a meaningful outcome.

When revising your manuscript:

* Include a "Response to reviewers" document detailing, point-by-point, how you addressed each referee comment. If no action was taken to address a point, you must provide a compelling argument. This response will be sent back to the reviewers along with the revised manuscript.

* Ensure that you use our template for Stage 1 Registered Reports to prepare your revised manuscript:
https://www.nature.com/documents/CP_Template-RR-Stage1.docx
Failure to ensure that your revised Stage 1 submission meets our requirements as specified in the template will result in your submission being returned to you, which will delay its consideration.

* In your cover letter, please include the following information:
--An anticipated timeline for completing the study if your Stage 1 submission is accepted in principle.
--A statement confirming that you agree to share your raw data, any digital study materials, computer code, and laboratory log for all eventually published results.
--A statement confirming that, following Stage 1 in principle acceptance, you agree to register your approved protocol on the Open Science Framework (https://osf.io/) or other recognised repository, either publicly or under private embargo, until submission of the Stage 2 manuscript.
--A statement confirming that if you later withdraw your paper, you agree to the Journal publishing a short summary of the pre-registered study under a section Withdrawn Registrations.

Please use the link below to submit your revised manuscript and related files:

Link Redacted

Note: This URL links to your confidential home page and associated information about manuscripts you may have submitted, or that you are reviewing for us. If you wish to forward this email to co-authors, please delete the link to your homepage.

We hope to receive your revised manuscript within four to eight weeks. If you cannot send it within this time, please let us know. We will be happy to consider your revision so long as the report still represents a significant contribution to the literature at that stage.

Please do not hesitate to contact me if you have any questions or would like to discuss these revisions further.

* TRANSPARENT PEER REVIEW: Communications Psychology uses a transparent peer review system. This means that we publish the editorial decision letters including Reviewers' comments to the authors and the author rebuttal letters online as a supplementary peer review file. We publish these records for all accepted manuscripts. However, on author request, confidential information and data can be removed from the published reviewer reports and rebuttal letters prior to publication. If your manuscript has been previously reviewed at another journal, those Reviewers' comments would not form part of the published peer review file.

Communications Psychology is committed to improving transparency in authorship. As part of our efforts in this direction, we are now requesting that all authors identified as 'corresponding author' on published papers create and link their Open Researcher and Contributor Identifier (ORCID) with their account on the Manuscript Tracking System (MTS), prior to acceptance. ORCID helps the scientific community achieve unambiguous attribution of all scholarly contributions. You can create and link your ORCID from the home page of the MTS by clicking on 'Modify my Springer Nature account'. For more information please visit www.springernature.com/orcid.

We look forward to seeing the revised manuscript and thank you for the opportunity to review your work.

Sincerely,

Troby Lui, PhD
Associate Editor
Communications Psychology

REVIEWERS' EXPERTISE:

Reviewer #1, #3, #4: craving/eating, RL/Pavlovian learning, ecological momentary assessment

Reviewer #2: craving/eating, approach-avoidance, experience sampling

REVIEWERS' COMMENTS:

Reviewer #1, #3, #4:
Remarks to the Author:
COMMSPSYCHOL-24-0736
We appreciate that the mobile AAT was used to boost the reliability for the implicit craving variable up to a level comparable with explicit craving measures.

Major comments:
Throughout this text, terms are used without introducing them first. A particularly important example is the regression coefficients on page 6. At this point in the text, nobody knows what w is yet. The authors should introduce their measures first before introducing how they will evaluate their hypotheses. Likewise, earlier in the intro the authors explain that learning biases probably play a big role in eating behavior, without explaining what learning biases are. That happens much later in the paragraph.
Regression coefficients tend to be unreliable as variables, which attenuates their correlation with other variables. H3 will thus probably be underpowered, since it is testing the difference between two likely small correlations, which will yield a very small difference. This may then require a large sample size. I would appreciate if the authors can report the reliability of this regression coefficient from previous research, and do a power analysis for this hypothesis. Otherwise, they may end up with a sample size that cannot answer this research question.
The approach-avoidance task measures approach-avoidance tendencies. It may be problematic to call this implicit craving - we cannot equate the behavioral tendency with an intense desire, we can only correlate it with that. If we are to follow dual-systems theories, approach-avoidance tendencies are merely stimulus-induced behavioral orientations without necessarily implying "wanting". At least a discussion is warranted.
Page 14: a clearer explanation of the computation of the w coefficient is needed here, this is not really something that can be relegated to the appendix.
Page 14: the approach-avoidance quotient employed here will probably give the authors a headache down the line since it has several undesirable properties: (1) a small value in the denominator can greatly inflate the value in the numerator, creating outliers, and relatedly, (2) the coefficient shifts in asymmetric amounts when the numerator or denominator shift - to illustrate, when the object bias is twice the food bias, the resulting score is 1/2 (hence 0.5 lower than 1), but when the food bias is twice the object bias, the resulting score is 2/1 (hence 1 higher than 1). This latter issue can be solved by taking the log() of the quotient. The former issue cannot really be solved though, so maybe a regular double difference score is better here. I recognize the authors employ robust outlier rejection here to guard against this issue, but given the use of moderation analyses and correlation comparisons, which tend to require bigger samples, it would probably be better to use methods that retain more participants in the sample.
Page 15, 16: The use of the standard deviation as an indicator of HCHP food craving is unexpected, unexplained, and as far as I can tell, not fitting for the questions the authors are trying to answer with these analyses.
Page 15: craving would also have to be a random slope here, since it is a within-person / level 1 variable. Leaving it out inflates the alpha error of this model above 0.05.
Minor comments:
Abstract: Please be explicit about whether the listed tasks will all be administered on the phone and in the manner of EMA (repetitive measures in daily life)
Introduction: it is not made clear what the role of prediction error is within model-free learning. An example would help
Hypothesis 3: "Therefore, we posit a stronger association between learning biases with either self-reported cravings or implicitly measured cravings." the words leading up to this sentence strongly suggest that there will be a stronger link with implicit cravings, so it is unexpected that this hypothesis is non-directional - how come?
Page 11: RT may need to be defined differently in the mobile AAT since participants are technically always moving the phone very small distances - some threshold will be needed.
How are "servings" defined (e.g., regarding candy or chocolate)? Is there a definition in the app for participants? Otherwise there might be some issue with the consumption measurement when e.g., a hamburger is treated the same as one chocolate piece as participants might indicate both as being "one serving" and both are classified as HCHP foods.
What is the maximum number of servings that can be indicated in the drop-down menu?
Line 229: Are the "various" food images included in the explicit liking rating all 129 images selected from food.pics?
At which time of the day will the notification for the mobileAAT task be sent?
Line 256: A word is missing here in the first sentence
Regarding the analysis part I am wondering whether it would be more reasonable for the analysis of hypothesis H2 to somehow take the different stimulus categories subsumed under HCHP foods into account (maybe by adding a random intercept for stimulus categories). That could solve the issue with the "serving" question I described above (and might also take into account the stimulus-specifity of cravings often assumed by learning theories)
Jens Blechert with the help of Sercan Kahveci and Mareike Roettger

Reviewer #2:
Remarks to the Author:
Dear authors,

Below is my review of your manuscript. I think the overall topic is interesting to learn more about reward learning in everyday life. I do have three points I think need the be addressed.

I think that the tasks presented and relations tested are mostly clear. What I struggled most with is to extract the exact purpose of the study. Is it to relate cognitive tasks to the ecological environment to validate these tasks or is it to examine basic learning processes in the ecological environment using validated tasks of reward learning? Either way, it seems imperative to discuss more the reliability and validity of the measures so that it becomes more clear how the relations can best be interpreted. Do we learn the tasks might be good proxies for learning in the ecological environment? Is this reasonable to assume? Why?
Relatedly, it is now unclear to me what are insights from the work beyond examining some relations. What do we learn from this study beyond the specific observed relations. other words, I think it would be helpful when the authors zoom out somewhat more and discuss the contribution from a broader perspective.
I think it is imperative that in a registered report like this hypotheses are clearly and unambiguously formulated and consistent throughout the text. This is now not the case. There is mentioning of theoretical hypotheses. What are these and can these be falsified djust as statistical hypothesis? For instance, on page 13 it is stated “H2) Learning biases might moderate the relationship between explicit cravings 364 and consumption of high-caloric high palatable (HCHP) foods”. Due to the word “might” this is an unfalsifiable hypothesis. The authors need to go carefully through the text and be very consistent in the formulation of hypotheses and make sure they can be tested and falsified.

Version 1:

Decision Letter:

** Please ensure you delete the link to your author homepage in this e-mail if you wish to forward it to your coauthors **

Dear Ms Tapparel,

Thank you for submitting your manuscript titled "From craving to consumption: the role of affective learning biases in daily food behaviour" to Communications Psychology. We have given the paper our careful consideration and find it of potential interest. However, due to certain shortcomings we are concerned that sending the current manuscript out to review could lead to unnecessary delays and quite possibly an undesirable outcome of the review process.

In particular, the revised manuscript fails to meet our requirements for the interpretation of null results. You currently preregister the intention to interpret non-significant findings as evidence for the absence of an association or effect, which is not permitted. Reporting a Bayes Factor alongside the main test does not resolve the issue, as the interpretation of the data must later follow the primary preregistered analyses. For instance, you write “If we fail to reject the null hypothesis for H1a, this would suggest that the conditioned motivational processes underlying cravings are not significantly modulated by individual differences in signtracking/goal-tracking tendencies, which would imply that the experienced mechanisms of cravings may be conserved across varying learning styles.”. The central claim of the conserved experienced mechanism of cravings cannot be supported by the failure to reject the null hypothesis, it requires positive evidence for the null hypothesis.

You need to preregister a Bayesian analysis or equivalence tests to be able to interpret a null finding. Given the research question and setup, we strongly recommend adopting Bayesian statistics and switching to a Bayesian sampling strategy (https://www.nature.com/commspsychol/submit/registered-reports). For Registered Reports, the minimum Bayes Factor to cease sampling is BF=10 (either in favour of against the H1), although it is permissible to also provide a feasibility criterion. If you were to adopt equivalence tests instead, we would ask for a revised smallest effect size of interest to render the potential null-results informative.

Regardless of whether you switch to continuous sampling with a feasibility opt-out or a predetermined sample size, the minimum number of participants needs to be set as the number maintained in the analyses post-exclusion, not the recruited sample. Please also preregister the gender ratio, which should aim for a more balanced distribution than the pilot data.

As we will not issue in principle acceptance for stage 1 Registered Reports without an ethics agreement in place, we strongly advise you to obtain ethics approval as soon as possible.

We would therefore like to invite you to revise your manuscript to address these concerns before we make a final determination on whether to send your manuscript for external review.

We shall hope to receive your revised version as soon as you are able to complete the suggested revisions. If something similar is published in the interim we will have to consider the impact it has on the novelty of a revised manuscript.

If you anticipate a delay of more than four weeks, please let us know. Should your manuscript be substantially delayed without notifying us in advance and your article is eventually published, the received date may be that of the revised, not the original, version.

We also ask that you ensure your manuscript complies with our editorial policies and reporting requirements.

To that end, we require revised manuscripts to be accompanied by two completed items: a reporting summary that collects information on study design and procedure, and an editorial policy checklist that verifies compliance with all required editorial policies.

- Nature Research Reporting Summary
- Editorial Policy Checklist

All points on the policy checklist must be addressed. Your revised manuscript can only be sent to referees if these checklists are completed and uploaded with the revision.

If you are not interested in submitting a suitably revised manuscript in the future please let me know immediately so we can close your file. If you have any questions, please contact me.

Please use the link below when you are prepared to resubmit.
Link Redacted

Thank you for your interest in Communications Psychology.

Best regards,
Troby Lui

Troby Lui, PhD
Associate Editor
Communications Psychology

Version 2:

Decision Letter:

Dear Ms Tapparel,

Thank you once again for your manuscript, entitled "From craving to consumption: the role of affective learning biases in daily food behaviour," and for your patience during the peer review process.

Your manuscript has now been evaluated by the same 4 reviewers as before. The reviewer reports confirm that the protocol is technically sound. However, the protocol at this stage falls short of meeting general and journal-specific requirements for Registered Reports. We will not be able to proceed to Stage 1 Acceptance in Principle until all of the remaining issues are entirely resolved.

The remaining issues pertain mostly to: ethics approval, choice of statistical tests, clarity and consistency of presentation, sampling strategy, and code availabilty. To aid you in the task of revising the work, I am attaching a guidance document for each section of the protocol with specific comments as to how the respective section needs to be revised.

* In your cover letter, please include the following information:
--An anticipated timeline for completing the study if your Stage 1 submission is accepted in principle.
--A statement confirming that you agree to share your raw data, any digital study materials, computer code, and laboratory log for all eventually published results.
--A statement confirming that, following Stage 1 in principle acceptance, you agree to register your approved protocol on the Open Science Framework (https://osf.io/) or other recognised repository, either publicly or under private embargo, until submission of the Stage 2 manuscript.
--A statement confirming that if you later withdraw your paper, you agree to the Journal publishing a short summary of the pre-registered study under a section Withdrawn Registrations.

Please use the link below to submit your revised manuscript and related files:

Link Redacted

Note: This URL links to your confidential home page and associated information about manuscripts you may have submitted, or that you are reviewing for us. If you wish to forward this email to co-authors, please delete the link to your homepage.

We hope to receive your revised manuscript within four to eight weeks. If you cannot send it within this time, please let us know. We will be happy to consider your revision so long as the report still represents a significant contribution to the literature at that stage.

Please do not hesitate to contact me if you have any questions or would like to discuss these revisions further.

* TRANSPARENT PEER REVIEW: Communications Psychology uses a transparent peer review system. This means that

we publish the editorial decision letters including Reviewers' comments to the authors and the author rebuttal letters online as a supplementary peer review file. We publish these records for all accepted manuscripts. However, on author request, confidential information and data can be removed from the published reviewer reports and rebuttal letters prior to publication. If your manuscript has been previously reviewed at another journal, those Reviewers' comments would not form part of the published peer review file.

Communications Psychology is committed to improving transparency in authorship. As part of our efforts in this direction, we are now requesting that all authors identified as ‘corresponding author’ on published papers create and link their Open Researcher and Contributor Identifier (ORCID) with their account on the Manuscript Tracking System (MTS), prior to acceptance. ORCID helps the scientific community achieve unambiguous attribution of all scholarly contributions. You can create and link your ORCID from the home page of the MTS by clicking on ‘Modify my Springer Nature account’. For more information please visit www.springernature.com/orcid.

We look forward to seeing the revised manuscript and thank you for the opportunity to review your work.

Sincerely,
Marike, on behalf of

Troby Lui

Marike Schiffer, PhD
Chief Editor
Communications Psychology

Troby Lui, PhD
Associate Editor
Communications Psychology

REVIEWERS' COMMENTS:

Reviewer #1:
Remarks to the Author:
The authors have responded well and in detailed manner to our comments and concerns. The registered report also sensibly deals with issues of power and null hypotheses in my view. So I recommend acceptance as is.

Reviewer #2:
Remarks to the Author:
I appreciate the careful responses by the authors to my comments.I think they handled them well, and I can thus recommend the manuscript for the next stage.

Reviewer #3:
Remarks to the Author:
I concur with the decision of my co-reviewer.

Reviewer #4:
None

Version 3:

Decision Letter:

20th Jun 2025

*Please ensure you delete the link to your author homepage in this e-mail if you wish to forward it to your co-authors.

Dear Ms Tapparel,

Thank you once again for submitting your revised Stage 1 Registered Report, entitled "Understanding the role of affective learning biases in daily food behaviour." Everything is in order and I am delighted to say that we can offer acceptance in principle. You may progress to Stage 2 and complete the study as approved.

As you know, a condition of in-principle-acceptance is that the authors agree to deposit their Stage 1 accepted protocol in a repository, either publicly or under embargo until Stage 2 acceptance and publication. We are very keen to showcase our in-principle accepted protocols, so that our readers, reviewers, and potential authors can gain insight into the requirements of the format as well as an idea of the types of projects that are suitable for publication in Communications Psychology. We have set up a space on figshare to host all of our in-principle accepted protocols, which can either be made public or kept under embargo until Stage 2 acceptance (depending on author preference). This gives you the opportunity to have your work publicly associated with Communications Psychology, and of course we will be very pleased to showcase your report if you agree to share it publicly.

Depositing the work on our figshare space does not preclude deposition of your Stage 1 protocol on other depositories – your protocol can also be posted on OSF, Dataverse, Dryad or any other public repository of your choice. You also do not need to do anything – if you agree with posting your protocol on our figshare space, we will upload your protocol on your behalf and either set it public or place it under embargo, depending on your choice. Your protocol will be licensed under a CC BY license (Creative Commons Attribution 4.0 International License). The CC BY license allows for maximum dissemination and re-use of open access materials and is preferred by many research funding bodies. Under this license users are free to share (copy, distribute and transmit) and remix (adapt) the contribution including for commercial purposes, providing they attribute the contribution in the manner specified by the author or licensor (read full legal code: http://creativecommons.org/licenses/by/4.0/legalcode) Please note that any use of https://springernature.figshare.com will be subject to the Figshare terms of use. Figshare has the right to enforce these terms and conditions where applicable. Use of third party services and sites will be subject to the relevant terms of use and will apply if we act on your behalf in this regard. Do let me know if you would like to take up this option or if you have any questions regarding the protocol deposition requirement.

IMPORTANT:
In cases where the registered experimental design is altered after AIP due to unforeseen circumstances (e.g. change of equipment or unanticipated technical error), the authors should consult the editors immediately for advice, prior to the completion of data collection.

Following completion of your study, we invite you to resubmit your paper for peer review as a Stage 2 Registered Report. Please note that your manuscript can still be rejected for publication at Stage 2 if the Editors consider any of the following to hold:

• The results were unable to test the authors' proposed hypotheses by failing to meet the approved outcome-neutral criteria
• The authors altered the Introduction, rationale, or hypotheses, as approved in the Stage 1 submission
• The authors failed to adhere closely to the registered experimental procedures without previously seeking editorial approval
• Any post hoc (unregistered) analyses were either unjustified, insufficiently caveated, or overly dominant in shaping the authors' conclusions
• The authors' conclusions were not justified given the data obtained

We encourage you to read the complete guidelines for authors concerning Stage 2 submissions at https://www.nature.com/commspsychol/submit/registered-reports and https://www.nature.com/documents/commspsychol-style-formatting-checklist-article-rr.pdf .

Please especially note the requirements for protocol deposition, data sharing, and that withdrawing your manuscript will result in publication of a Retracted Registration.

When you are ready, please use the following link to access your home page and submit your Stage 2 Registered Report:

Link Redacted

*This url links to your confidential homepage and associated information about manuscripts you may have submitted or be reviewing for us. If you wish to forward this e-mail to co-authors, please delete this link to your homepage first.

* TRANSPARENT PEER REVIEW: Communications Psychology uses a transparent peer review system. This means that we publish the editorial decision letters including Reviewers' comments to the authors and the author rebuttal letters online as a supplementary peer review file. This means that the records will be published together with your Stage 2 report. On author request, confidential information and data can be removed from the published reviewer reports and rebuttal letters prior to publication. If your manuscript has been previously reviewed at another journal, those Reviewers' comments would not form part of the published peer review file.

We expect your Stage 2 Registered Report to be submitted by the date specified in your latest cover letter. If unforeseen circumstances prevent submission by that date, please contact us as soon as possible to discuss any changes to the submission time-frame.

Thank you again for offering us this work and we look forward to receiving your Stage 2 Registered Report.

Yours sincerely,

Troby Lui, PhD
Associate Editor
Communications Psychology

Version 4:

Decision Letter:

24th Jun 2026

*Please ensure you delete the link to your author homepage in this e-mail if you wish to forward it to your co-authors.

Dear Ms Tapparel,

Thank you once again for submitting your Stage 2 Registered Report, entitled "Understanding the role of affective learning biases in daily food behaviour," and for your patience during the re-review process.

Your manuscript has now been evaluated by Reviewers [1,2,3,and 4] from the previous rounds of review, whose comments are included at the end of this letter. In the light of our reviewers' advice, we are pleased to inform you that we will be able accept your Stage 2 manuscript, pending revisions to address Reviewers' comments and editorial requests.

Please address the remaining suggestions raised by the two reviewers, in particular regarding the additional elaboration on the floor effect and the rationale for the cease of data collection in the main text.

Note that while the pilot study must be placed in the supplement, all other decision-relevant analyses, such as the floor effect analysis (Section 6 in the supplementary information), should be in the main text. Please keep the sentence "We conducted a pilot analysis to assess the feasibility and interpretability of the measures used in this protocol" and include a reference to the supplementary information.

One of the main reasons for delays in eventual acceptance is failure to fully comply with editorial policies and formatting requirements. To assist you with finalizing your manuscript for publication, I attach our Editorial Requests Table which lists all of our editorial policies and formatting requirements.

Please attend to *every item* in the Table and upload a copy of the completed checklist with your submission. I have highlighted in the checklist items that require your attention. I also mention here a few points that are frequently missed and can cause delays:

1) Please insert the following Protocol Registration information in your manuscript:

The Stage 1 protocol for this Registered Report was accepted in principle on June 20th, 2025. The protocol, as accepted by the journal, can be found at https://doi.org/10.6084/m9.figshare.29445518.

2) Ensure that all corresponding authors have linked their ORCID to their account on our online manuscript handling system. This is very frequently missed and invariably causes delays in formal acceptance.

3) Ensure that you provide all of the materials requested in the attached checklist and below with your final submission.

OPEN ACCESS:

Communications Psychology is a fully open access journal. Articles are made freely accessible on publication. For further information about article processing charges, open access funding, and advice and support from Nature Research, please visit https://www.nature.com/commspsychol/open-access

At acceptance, you will be provided with instructions for completing the open access licence agreement on behalf of all authors. This grants us the necessary permissions to publish your paper. Additionally, you will be asked to declare that all required third party permissions have been obtained, and to provide billing information in order to pay the article-processing charge (APC).

* TRANSPARENT PEER REVIEW: Communications Psychology uses a transparent peer review system. On author request, confidential information and data can be removed from the published reviewer reports and rebuttal letters prior to publication. If you are concerned about the release of confidential data, please let us know specifically what information you would like to have removed. Please note that we cannot incorporate redactions for any other reasons.

* CODE AVAILABILITY: To proceed to formal acceptance, you must now publicly deposit the custom analysis code supporting your conclusions; please use a repository that mints the code with a digital object identifier (DOI). The manuscript must include a section titled "Code Availability" at the end of the methods section. The link to the repository and the DOI must be included in the Code Availability statement. Publication as Supplementary Information will not suffice.

* DATA AVAILABILITY:
It is a requirement for Registered Reports to make data publicly available. All Communications Psychology manuscripts must include a section titled "Data Availability" at the end of the Methods section. More information on this policy, is available in the Editorial Requests Table and at http://www.nature.com/authors/policies/data/data-availability-statements-data-citations.pdf. Please share a link to your publicly deposited data in the Data Availability statement.

We hope to hear from you within [ENTER TIME PERIOD]; please let us know if the revision process is likely to take longer.

Please use the following link for uploading the materials:
Link Redacted

If you have any further questions, please feel free to contact me.

With best regards,

Troby Lui, PhD
Associate Editor
Communications Psychology

Reviewer #1:
Remarks to the Author:
The MS is very ripe now. I recomment acceptance pending minor revisions.

The authors provide a rationale for why implicitly measured cravings might be more strongly associated with learning biases. However, since they formulate a non-directional hypothesis, it would be helpful if they could also explain why learning biases might instead be more strongly associated with explicitly measured cravings.

Please note that the paper cited by the authors for the AAT procedure has since been retracted. However, another paper describes the same study and therefore the same procedure: https://doi.org/10.1016/j.appet.2026.108462

Reviewer #2:
Remarks to the Author:
This is a very well executed study. I focused on the blue (new parts) of the report. I think the authors did a very good job of being very nuanced in their conclusions so I do not have any major points, only a few minor points:

Abstract is very technical. Consider to write it somewhat more accessible without jargon.

It would further be important to add some of the main possible causes for the null effects to the abstract including the healthy sample and the floor effect on the consumption measure.

Why is the pilot study omitted from the current manuscript?

This argument requires some additional elaboration. “Due to the floor effect observed for H2 (See Supplementary Material for detailed analysis), the final sample target was revised to match that of H1 and H3. Recruitment continued until the post-exclusion sample included 63 participants with complete datasets. Distribution outliers were not planned to be replaced to avoid circularly changing the thresholds for exclusion.”
I understand that it is in the supplements, but still it would be good to explain this a little in the main text. Specifically, when was descried that there is a floor effect and that additional sampling would not address this?

Table 2 can be replaced by text.

In the conclusion it is stated: “In contrast, the positive association between model-free bias and craving provides preliminary evidence for the role of habit-like reinforcement processes in everyday eating.” Since there is no action at all in the consumption measure I would rephrase the everyday eating part.

Maybe reflect on the limitations of Bayes factors for quantifying null findings.

Reviewer #3:
Remarks to the Author:
I have no objections to the publication of this manuscript.

Reviewer #4:
Remarks to the Author:
I co-reviewed this manuscript with one of the reviewers who provided the listed reports. This is part of the Communications Psychology initiative to facilitate training in peer review and to provide appropriate recognition for Early Career Researchers who co-review manuscripts.

Open Access This Peer Review File is licensed under a Creative Commons Attribution 4.0 International License, which permits use, sharing, adaptation, distribution and reproduction in any medium or format, as long as you give appropriate credit to the original author(s) and the source, provide a link to the Creative Commons license, and indicate if changes were made.
In cases where reviewers are anonymous, credit should be given to 'Anonymous Referee' and the source.
The images or other third party material in this Peer Review File are included in the article’s Creative Commons license, unless indicated otherwise in a credit line to the material. If material is not included in the article’s Creative Commons license and your intended use is not permitted by statutory regulation or exceeds the permitted use, you will need to obtain permission directly from the copyright holder.
To view a copy of this license, visit https://creativecommons.org/licenses/by/4.0/

Answers to reviewers

Our reply to each of the comment are reported in blue, and the text that has been modified in the manuscript reported in green.

REVIEWERS' EXPERTISE:

Reviewer #1, #3, #4: craving/eating, RL/Pavlovian learning, ecological momentary assessment

Reviewer #2: craving/eating, approach-avoidance, experience sampling

REVIEWERS' COMMENTS:

Reviewer #1, #3, #4:
Remarks to the Author:
COMMSPSYCHOL-24-0736
We appreciate that the mobile AAT was used to boost the reliability for the implicit craving variable up to a level comparable with explicit craving measures.

Major comments:

1. Throughout this text, terms are used without introducing them first. A particularly important example is the regression coefficients on page 6. At this point in the text, nobody knows what w is yet. The authors should introduce their measures first before introducing how they will evaluate their hypotheses.

We thank the reviewer for the remark. Mentions of the regression coefficient & w in the hypothesis were removed and those were further explained in the last paragraph p.7. It reads now: "The Pavlovian bias is indexed as the regression coefficient of a linear regression predicting the gaze direction from the reward value during a Pavlovian conditioning task. To evaluate reinforcement learning bias, we will use the weighting parameter *w* indexing the relative contribution of model-free and model-based systems in the net value of the action in a sequential learning task."

2. Likewise, earlier in the intro the authors explain that learning biases probably play a big role, without explaining what learning biases are. That happens much later in the paragraph.

The paragraph has been restructured to define learning biases earlier in the text. The definition reads p.4 as: "Learning mechanisms in Pavlovian and reinforcement learning explain how environmental cues gradually acquire motivational value and, in turn, influence consumption choices. In Pavlovian learning, palatable food items (and the related cues) become associated with both central (e.g., brain reward circuitry) and peripheral (e.g., salivation, digestive readiness) responses. These responses may reflect pleasure-related physiological reactions and preparatory mechanisms for food consumption. In reinforcement learning, values are attributed to actions in response to food cues. Biases in those learning mechanisms represent a relevant framework to understand cravings and their link to consumption because they influence how people respond to food cues, ultimately

affecting eating behaviours. In Pavlovian learning, individuals biased toward sign-tracking attribute incentive value to the cue itself, and goal-trackers focus on the cue-outcome predictive relationship. Attentional bias towards food cues - an indirect measure of cues' incentive salience in sign-trackers - has been consistently linked with tendencies for binge eating and food cravings. In reinforcement learning, individuals are biased toward model-free, habitual learning updating action values directly from reward prediction errors such as associating a positive value with a favorite coffee shop due to consistently rewarding past experiences, whereas model-based, goal-directed learners build up internal models of the environment to predict outcomes, such as estimating the value of different coffee options considering factors like cost, time, and health benefits. A greater reliance on model-free learning has been reported in patients with obesity and binge eating disorders compared to healthy controls."

3. Regression coefficients tend to be unreliable as variables, which attenuates their correlation with other variables. H3 will thus probably be underpowered, since it is testing the difference between two likely small correlations, which will yield a very small difference. This may then require a large sample size. I would appreciate if the authors can report the reliability of this regression coefficient from previous research, and do a power analysis for this hypothesis. Otherwise, they may end up with a sample size that cannot answer this research question.

We agree. Given the expected small size of the difference in correlation tested in H3 and the nature of the regression coefficient dependent variable, we would need 275 data points to reach a 95% power to detect it at an alpha of 0.05, which would exceed the resources available for this study. We thus propose to remove this comparison from the preregistration (though exploring it in the Discussion section of the stage 2), and to replace it in the registration plan a more direct hypothesis on the correlation between approach bias and learning biases.

Accordingly, the **Introduction section** now reads, p.5

1) *Learning biases are associated with approach bias*

Approach bias is defined as the tendency to respond more quickly when approaching rather than avoiding food stimuli and is thought to originate in part from automatic processes. In the context of eating behaviour, approach bias is a direct measure of the automatic processes involved in craving formation and is assumed to drive consumption behaviour, it may thus be associated with sign-tracking bias as they both capture automatic processes.

Lastly, given that sign-tracking biases and approach bias both capture automatic processes, they may share a more direct connection with approach bias compared to self-reported cravings. However, while implicit measures are theoretically designed to capture automatic processes, self-reported measures may better reflect the subjective reality of participants[32] and align with participants' lived experiences or preferences. Therefore, we posit a stronger association between learning biases and either approach bias or self-reported cravings."

and p.6: "

- H3) Pavlovian/reinforcement learning biases are correlated with approach bias for HCHP foods (Figure 1, Table 1; H3); We will test this hypothesis by analysing the correlation between Pavlovian bias and approach bias (H3a). We will then analyse the correlation

between reinforcement learning bias and approach bias (H3b). The hypothesised mechanisms of action stated above lead us to expect an effect between approach bias and sign-tracking, which might not be observed between approach bias and reinforcement learning bias. Moreover, we will use a descriptive approach to test whether Pavlovian/reinforcement learning biases are more strongly correlated with either approach bias or self-reported for HCHP foods, comparing the correlation between learning biases and these two measures (i.e., self-reported cravings and approach bias, respectively)."

Analysis plan p. 15:

H3) Learning biases are correlated with approach bias for HCHP foods

We will compute the correlation between the approach bias for HCHP foods and the learning biases (H3a: Pavlovian bias, H3b: reinforcement learning bias). To capture both inter- and intra-individual variability, the approach bias for HCHP foods will be defined as i) the mean of approach biases and ii) the standard deviation of approach biases over the two-week study period. Using both measures enables a comprehensive assessment of the approach bias dynamics: while the mean reflects the general approach bias tendency, the standard deviation captures the magnitude of fluctuations over time. As such, two correlations will be computed for both H3a and H3b. A Bonferroni correction will be applied to account for multiple testings (alpha of 0.025). We hypothesise that Pavlovian bias will be correlated with approach bias for HCHP foods (H3a). While still wanting to investigate a potential relationship between reinforcement learning bias and approach bias for HCHP foods (H3b), we are less confident that it exists (see Introduction).

We will report the Bayes Factor (BF_{01}) alongside p-values, where a value above 3 will indicate evidence supporting the absence of a significant relationship between the relevant learning bias and approach bias.

Additionally, as a descriptive secondary endpoint, we will compare correlations between both self-reported cravings and approach biases for both learning biases. To capture both inter- and intra-individual variability, we will use two measures: (i) the mean daily cravings or approach biases and (ii) the standard deviation of daily cravings or approach biases over the two-week study period. We expect a difference of r of approximately 0.2 between the self-reported and approach bias correlations for at least one outcome (i.e., mean or standard deviation), which would be sufficient to shift the effect size classification (e.g., from small to moderate). A higher correlation in approach bias would suggest learning biases are more directly linked to the captured automatic processes, whereas a higher correlation in self-reported cravings would indicate that learning biases are more closely linked to subjective experience rather than automatic processes. Differences in correlation for the mean would suggest one measure captures craving intensity better, while differences in the standard deviation would point to differences in capturing craving variability. If no difference of at least 0.2 is observed, this would imply that both measures capture similar aspects of the relationship or that the link between cravings and learning biases is too weak to distinguish between them."

Sampling plan p. 16: "*H3) Learning biases are correlated with approach bias for HCHP foods*

For both Pavlovian bias (H1a) and reinforcement learning bias (H1b), we determine an SESOI to be a correlation of 0.4 (small to medium effect) for the association between learning biases and approach bias. A-priori power analysis conducted with the pwr R package with an alpha of .05 indicates that 63 participants are required to reach a power of .95 for a one-sided correlation.”

As well as in the **Design table** p.25:

Are learning biases correlated with approach bias?	H3a: Pavlovian biases are correlated with approach bias for HCHP foods	63 participants based on a priori power analysis with an r of 0.4, alpha of 0.05 and power of 0.95<br><br>Will be excluded during analyses:<br><br>- participants with error rate above 10% at the Forced Choice task	Two correlations with 1) mean of approach biases for HCHP foods, and 2) standard deviation of approach biases for HCHP foods for both (H1a: regression coefficient of CS value on the eye-gaze index for Pavlovian Learning; H1b: w index for SL) with an alpha of 0.025 to account for multiple testing.	If $p<0.025$ (Bonferroni-adjusted significance level) on at least one correlation, then the Pavlovian bias is significantly associated with higher approach bias for HCHP foods. A mean difference would suggest that individuals with stronger Pavlovian biases tend to exhibit a generally higher tendency to approach HCHP foods, indicating a shift in overall approach bias. A difference in standard deviation would suggest that Pavlovian biases are associated with greater variability in approach behaviour towards HCHP foods.<br><br>If $p \geq 0.025$, we will conclude that we cannot reject the null hypothesis for H3a.<br><br>We will additionally report the Bayes Factor (BF_{01}) alongside p-values, where a value above 3 will indicate evidence supporting the absence of a significant relationship between Pavlovian biases and approach bias.

	H3b: model-free biaises are correlated with approach bias for HCHP foods	63 participants based on a priori power analysis with an r of 0.4, alpha of 0.05 and power of 0.95<br><br>Will be excluded during analyses:<br><br>- participants with more than 20% of excluded trials<br><br>- participants wih a probability of stay when common rewarded trial worse than chance		If p<0.025 (Bonferroni-adjusted significance level) on at least one correlation, then the reinforcement learning bias is significantly associated with higher approach bias for HCHP foods. A mean difference would suggest that model-free bias directly or indirectly contributes to a higher overall tendency to approach HCHP foods. A standard deviation difference would suggest that model-free bias directly or indirectly influences the variability in approach behaviour.<br><br>If p≥0.025, we will conclude that we cannot reject the null hypothesis for H3a.<br><br>We will additionally report the Bayes Factor (BF_{01}) alongside p-values, where a value above 3 will indicate evidence supporting the absence of a significant relationship between reinforcement learning biases and approach bias.

The revised power analysis script was uploaded in the project's OSF page.

Concerning the reliability of regression coefficient as a measure. To the best of our knowledge and as mentioned in e.g. Colaizzi et al., 2020, no studies have directly evaluated the reliability of the regression coefficient-derived sign-tracking bias, but we have now added a "Pilot data" section in the manuscript. This pilot analysis shows that our values for the regression coefficient fall within the range of the existing literature (Schad et al., 2020). Of note, this literature has identified associations between these measures and acquired incentive saliency, thereby supporting the reliability, or at least the functional relevance of these measures (Pbootstrap < 0.05; b = 0.49; s.e. = 0.26; 95% CI = 0.09 to infinity).

4. The approach-avoidance task measures approach-avoidance tendencies. It may be problematic to call this implicit craving - we cannot equate the behavioral tendency with an intense desire, we can only correlate it with that. If we are to follow dual-systems theories, approach-avoidance tendencies are merely stimulus-induced behavioral orientations without necessarily implying "wanting". At least a discussion is warranted.

We agree that the term 'wanting' was misleading to refer to our measure of approach tendency, and thus now use only approach bias to refer to the measure of the approach-avoidance task, and have adjusted the paragraph p.5 in the **Introduction** section to introduce the concept:

"*3) Learning biases are associated with approach bias*

Approach bias is defined as the tendency to respond more quickly when approaching rather than avoiding food stimuli and is thought to originate in part from automatic processes. In the context of eating behaviour, approach bias is a direct measure of the automatic processes involved in craving formation and is assumed to drive consumption behaviour, it may thus be associated with sign-tracking bias as they both capture automatic processes.

Lastly, given that sign-tracking biases and approach bias both capture automatic processes, they may share a more direct connection with approach bias compared to self-reported cravings. However, while implicit measures are theoretically designed to capture automatic processes, self-reported measures may better reflect the subjective reality of participants[32] and align with participants' lived experiences or preferences. Therefore, we posit a stronger association between learning biases and either approach bias or self-reported cravings."

5. Page 14: a clearer explanation of the computation of the w coefficient is needed here, this is not really something that can be relegated to the appendix.

The paragraph was developed to include details of the w coefficient computation. It reads now, p. 11: "Reinforcement learning computational modeling will be used to calculate the reinforcement learning bias during the first-stage action (i.e., boat selection). The pure model-free value is computed using a temporal difference (TD) algorithm, updating action values based solely on received rewards from previous trials, whereas the model-based value incorporates the boat-island transition probabilities in its estimation of action value. The value of the participant's actual first-stage choice (Qnet) is a weighted combination of the model-based and model-free computational models ($Qnet = wQMB + (1\text{-}w)QTD$)). The weighting parameter w of this equation will be used as the index of the learning bias. A low w indicates a stronger reliance on the model-free strategy (Supplementary materials for detailed computations)."

6. Page 14: the approach-avoidance quotient employed here will probably give the authors a headache down the line since it has several undesirable properties: (1) a small value in the denominator can greatly inflate the value in the numerator, creating outliers, and relatedly, (2) the coefficient shifts in asymmetric amounts when the numerator or denominator shift - to illustrate, when the object bias is twice the food bias, the resulting score is 1/2 (hence 0.5 lower than 1), but when the food bias is twice the object bias, the resulting score is 2/1 (hence 1 higher than 1). This latter issue can be solved by taking the log() of the quotient. The former issue cannot really be solved though, so maybe a regular double difference score is better here.

We thank the reviewer for these insights and constructive advices. We have now changed the computation to the double difference score. It reads now, in p.13: "The approach bias will be measured every other day and will be computed as a double-difference score as in van Alebeek et al., 2024, by subtracting the difference between the session averaged reaction time for approach of HCHP and objects from the difference between the session averaged reaction time for avoidance of HCHP and objects.

Approach bias = ([HCHP avoidance] - [HCHP approach]) - ([Object avoidance] - [Object approach])"

7. I recognize the authors employ robust outlier rejection here to guard against this issue, but given the use of moderation analyses and correlation comparisons, which tend to require

bigger samples, it would probably be better to use methods that retain more participants in the sample.

Again, we thank the reviewers for their suggestion and have now increased the outlier distribution threshold to 3*MAD instead of 2.5*MAD (less conservative estimate; p 13), which is considered a standard conservative value for outlier exclusion (Leys et al., 2013). Excluding distribution outliers should remove participants not complying with the tasks or whose values fall outside theoretically plausible or expected ranges based on prior literature or established criteria. In our view, increasing the sample size to reduce the p-value is less desirable than interpreting a dataset reflecting a reliable population.

8. Page 15, 16: The use of the standard deviation as an indicator of HCHP food craving is unexpected, unexplained, and as far as I can tell, not fitting for the questions the authors are trying to answer with these analyses.

While we acknowledge the point raised by the reviewer, we think that the inclusion of the standard deviation alongside the mean would allow us to capture aspects of craving dynamics that the mean alone could not reflect. Specifically, while the average craving level indexes the general tendency or average intensity of cravings, the standard deviation reflects the amplitude of the fluctuations in craving intensity over time. This measure could be important because individuals might maintain a stable average level of cravings (unchanged mean) but experience significant fluctuations in intensity (increased standard deviation), or the reverse. Hence, examining the standard deviation allows us to differentiate between shifts (captured by the mean) and variability in cravings (captured by the standard deviation).

- A change in the mean suggests a shift in craving intensity (an overall higher or lower tendency to crave).
- A change in the standard deviation suggests a shift in craving variability (an overall higher or lower span of craving intensity), which might reflect different underlying processes than the average craving intensity, such as context sensitivity or reactivity to cues.

This justification has been added in the Analysis plan section and now reads p.15: "Using both measures enables a comprehensive assessment of craving dynamics: while the mean reflects the general tendency or average intensity of cravings, the standard deviation captures the magnitude of fluctuations in craving intensity."; and the Design table has been modified to include those interpretations and now reads p.23 (H1): "A mean difference would suggest a shift in craving intensity. A standard deviation difference would suggests a shift in craving variability." and p.22 (H3): "A mean difference would suggest that individuals with stronger Pavlovian biases tend to exhibit a generally higher tendency to approach HCHP foods, indicating a shift in overall approach bias. A difference in standard deviation would suggest that Pavlovian biases are associated with greater variability in approach behaviour towards HCHP foods."

9. Page 15: craving would also have to be a random slope here, since it is a within-person / level 1 variable. Leaving it out inflates the alpha error of this model above 0.05.

We thank the reviewer for his attention on this, the mistake has been corrected. It reads now, p. 15: "(i.e., consumption ~ cravings * learning bias + (cravings|participant))."

10. Abstract: Please be explicit about whether the listed tasks will all be administered on the phone and in the manner of EMA (repetitive measures in daily life)

The sentence was adjusted to reflect those changes: "To test these hypotheses, a two-week longitudinal study will use Ecological Momentary Assessments through a smartphone-based app to measure high-calorie food cravings and intake, as well as approach-avoidance in real-world contexts."

11. Introduction: it is not made clear what the role of prediction error is within model-free learning. An example would help

We thank the reviewer for this comment. The following exemples were added for more clarity, p.4: "In reinforcement learning, individuals are biased toward model-free, habitual learning updating action values directly from reward prediction errors such as associating a positive value with a favorite coffee shop due to consistently rewarding past experiences, whereas model-based, goal-directed learners build up internal models of the environment to predict outcomes, such as estimating the value of different coffee options considering factors like cost, time, and health benefits."

12. Hypothesis 3: "Therefore, we posit a stronger association between learning biases with either self-reported cravings or implicitly measured cravings." the words leading up to this sentence strongly suggest that there will be a stronger link with implicit cravings, so it is unexpected that this hypothesis is non-directional - how come?

We thank the reviewer for this remark. We have revised Hypothesis 3 (see comment #3), which indirectly addresses this concern, making the original issue no longer relevant. The previous H3 is now a descriptive secondary endpoint but has still been adjusted to reflect its non-directionality which now reads p.5: "Lastly, given that sign-tracking biases and approach bias both capture automatic processes, they may share a more direct connection with approach bias compared to self-reported cravings. However, while implicit measures are theoretically designed to capture automatic processes, self-reported measures may better reflect the subjective reality of participants and align with participants' lived experiences or preferences. Therefore, we posit a stronger association between learning biases and either approach bias or self-reported cravings."

13. Page 11: RT may need to be defined differently in the mobile AAT since participants are technically always moving the phone very small distances - some threshold will be needed.

It is indeed unclear, the definition was adjusted p. 11: "The reaction time (RT) is measured as the time from picture to movement onset."

14. How are "servings" defined (e.g., regarding candy or chocolate)? Is there a definition in the app for participants? Otherwise there might be some issue with the consumption measurement when e.g., a hamburger is treated the same as one chocolate piece as participants might indicate both as being "one serving" and both are classified as HCHP foods.

We agree with the reviewer and thank him for this insightful comment! To address this issue, we have now refined our definition of consumption by removing the use of servings and computing eating events, close to the methodology of Roefs et al. (2019). However, we would still ask participants about the amount of servings to implement a threshold of 200 kcal to define an eating event. This threshold was chosen as it reflects a typical snack size for HCHP foods and allows us to capture meaningful eating episodes without being influenced by very small snacks. Participants will be extensively instructed about each categories' serving sizes at the beginning of the study.

For example, under this new definition, eating a hamburger followed by an ice cream for dessert would count as two separate eating events. We believe that this approach will provide a more accurate representation of consumption patterns without introducing the bias that could result from having to standardize serving sizes for diverse food types.

Those changes were reflected in p. 10 which now reads: "Finally, participants will report the quantity of food consumed by answering, "How many servings of food from this category did you have?" This question will display the corresponding food icon alongside a drop-down menu, allowing participants to select the number of servings. Participants will receive extensive instructions about the definition of servings for each category at the beginning of the study. Daily self-reported consumption will be computed as the number of HCHP food consumption events**,** defined as eating events within a category that exceed a minimum of 200 kcal - a threshold reflecting a typical snacking event for HCHP foods (e.g., 1 croissant ≈ 230 kcal, 1 small ice cream cup ≈ 200 kcal)"; and in p. 13 which now reads: "The self-reported consumption will be measured every day and will be computed as the number of HCHP food consumption events**,** defined as eating events within a category that exceed a minimum of 200 kcal - a threshold reflecting a typical snacking event for HCHP foods (e.g., 1 croissant ≈ 230 kcal, 1 small ice cream cup ≈ 200 kcal).

Daily consumption= ∑HCHP consumption event/∑daily assessments"

15. What is the maximum number of servings that can be indicated in the drop-down menu?

The aim of the drop-down menu is to be unbounded; however, for technical feasibility, it will go up to 100 servings, which should accommodate the vast majority of use cases.

16. Line 229: Are the "various" food images included in the explicit liking rating all 129 images selected from food.pics?

Yes, for clarity, the term "various" was removed in p.10.

17. At which time of the day will the notification for the mobileAAT task be sent?

This was indeed unclear in the manuscript. The mobile AAT notification will be sent 1-hour before dinner time, which is set by the participant at the start of the experiment. This was clarified p.10: "To assess food approach bias in daily life, we will use a homemade Mobile AAT adapted from the version developed by Zech et al. (2023), with the procedure based on van Alebeek et al., 2024. Participants will receive a notification prompting them to complete the mobile-AAT task every two days, 1-hour before dinner time, resulting in seven individual assessments. Participants can customise their usual dinner time at the beginning of the experiment."

18. Line 256: A word is missing here in the first sentence

Indeed, we have now corrected the sentence to: "The Pavlovian conditioning task will **consist** of 80 trials, in which five fractal images (Conditional Stimulus; CS) are associated with five different reward values (Unconditional Stimulus; US)".

19. Regarding the analysis part I am wondering whether it would be more reasonable for the analysis of hypothesis H2 to somehow take the different stimulus categories subsumed under HCHP foods into account (maybe by adding a random intercept for stimulus categories). That could solve the issue with the "serving" question I described above (and might also take into account the stimulus-specifity of cravings often assumed by learning theories)

We thank the reviewer for this suggestion. While we agree that cravings are relatively stimulus-specific, we believe that restricting the analysis to individual HCHP items would be too limiting. For example, a craving for cake leading to eating a croissant is a common pattern of craving resolution that could be missed. We also considered using broader categories (e.g., sweet treats), but were concerned that this might introduce subjective bias in the categorization process. We believe that focusing on HCHP instead of all foods, is already a form of category restriction and would be sufficient in this regard.

Jens Blechert with the help of Sercan Kahveci and Mareike Roettger

---

Reviewer #2:
Remarks to the Author:
Dear authors,

Below is my review of your manuscript. I think the overall topic is interesting to learn more about reward learning in everyday life. I do have three points I think need to be addressed.

20. I think that the tasks presented and relations tested are mostly clear. What I struggled most with is to extract the exact purpose of the study. Is it to relate cognitive tasks to the ecological environment to validate these tasks or is it to examine basic learning processes in the ecological environment using validated tasks of reward learning?

We thank the reviewer for this feedback. The objective is indeed to examine basic learning processes in the ecological environment using validated tasks of reward learning. The study does not aim primarily at validating cognitive tasks themselves. Rather, we aim to understand how learning biases relate to eating behaviour in everyday contexts. By focusing on eating behaviour in an ecological environment, we seek to capture how cues and actions present in participants' natural settings influence their behaviour, providing more ecologically valid insights into the mechanisms underlying food-related decision-making, this explanation was added to the manuscript p.7: "These findings will enhance our understanding of the mechanisms driving food-related decision-making in daily life by

assessing whether learning biases can effectively characterise cravings and consumption, establishing learning bias tasks as tools to identify behaviours that may lead to maladaptive eating patterns and related pathologies (i.e obesity, type 2 diabetes)"

21. Either way, it seems imperative to discuss more the reliability and validity of the measures so that it becomes more clear how the relations can best be interpreted. Do we learn the tasks might be good proxies for learning in the ecological environment? Is this reasonable to assume? Why?

We appreciate the reviewer's feedback on the reliability and validity of our measures. Measuring consumption and cravings has indeed historically been challenging in the field of eating behaviour. EMA offers a more precise and context-sensitive approach by capturing participants' experiences in real-time and in their natural environments, reducing recall bias and enhancing ecological validity. A few validation studies have consistently showed that EMA improved the assessment of eating behaviour (Maugeri & Barchitta, 2019). Bucher Della Torre et al., 2017, for instance assessed EMA compared to traditional measure of consumption and showed 1) good usability and acceptability, 2) comparable nutrient precision (good correlation between EMA measured and traditional method, 3) precision of food intake compared to actual intake (77.3% vs. 60.0% of exact matches with the actual food intake). A justification was added in the manuscript p.6, which now reads: "The EMA approach involves collecting data in real-time and in participants' natural environments, enhancing ecological validity and minimising response bias and confounds related to retrospective memory deformations. This approach has shown to be an effective method for capturing the specificity, intensity, and diversity of food cravings and consumption in real time, providing insights that would not be possible through retrospective measures (Aulbach et al., 2025 , with Jens Blechert as last author and reviewer of this manuscript;; Roefs et al., 2019) "

The justification for the use of the Mobile-AAT instead of single implicit measure is mentioned in p.7: "This method improves the reliability issues commonly associated with single-measure implicit assessments, such as temporal fluctuations in bias scores and their covariation with factors like affect or hunger."

Concerning Pavlovian and reinforcement learning tasks, their robustness has been consistently demonstrated, e.g. Colaizzi et al., 2020; Daw et al., 2011; Pearce et al., 2022; Schad et al., 2020; Voon et al., 2015, 2017, and now recognised as the standard approach for capturing learning biases. Given their extensive use and empirical support, we consider these tasks provide a reliable and valid measure of learning biases. This argument was added p.7, which now reads: " The use of Pavlovian and sequential learning tasks is well-established in the literature and those tasks are recognised as the standard approach for capturing learning biases."

In the present study, those tasks are used to measure participants' pre-existing learning profiles, which we posit influence how we perceive cues and decide on actions in everyday life. By linking these tasks with EMA measures, we will understand how these biases interact with real-time cues and actions to influence daily states and choices.

22. Relatedly, it is now unclear to me what are insights from the work beyond examining some relations. What do we learn from this study beyond the specific observed relations. other

words, I think it would be helpful when the authors zoom out somewhat more and discuss the contribution from a broader perspective.

We agree with the reviewer's comment. Beyond examining specific relationships between learning biases, approach bias, and cravings, the study aims to clarify the mechanisms through which cravings influence eating behaviours. These insights could inform the development of more targeted interventions for eating disorders and inspire further research on the links between affective learning, cravings, and food-related behaviours.

This rationale was added to the manuscript p.7: "These findings will enhance our understanding of the mechanisms driving food-related decision-making by assessing whether learning biases can effectively characterise cravings and by establishing learning bias tasks as tools to identify behaviours that may lead to maladaptive eating patterns and related pathologies (i.e., obesity, type 2 diabetes; Farr & Mantzoros, 2018; Makaronidis & Batterham, 2018; Reents & Pedersen, 2021; Stice et al., 2015). Additionally, integrating this new dimension of eating behaviour could inform the development of personalised solutions for eating behaviour by increasing the precision and efficacy of existing interventions such as response training(Najberg et al., 2021, 2023) "

23. I think it is imperative that in a registered report like this hypotheses are clearly and unambiguously formulated and consistent throughout the text. This is now not the case. There is mentioning of theoretical hypotheses. What are these and can these be falsified djust as statistical hypothesis? For instance, on page 13 it is stated "H2) Learning biases might moderate the relationship between explicit cravings 364 and consumption of high-caloric high palatable (HCHP) foods". Due to the word "might" this is an unfalsifiable hypothesis. The authors need to go carefully through the text and be very consistent in the formulation of hypotheses and make sure they can be tested and falsified.

Agreed. We thank the reviewer for raising this point. The hypothesis were made clear and consistent (p. 6, 14,15, Design table); except for the Introduction section (p.4 &5) where the word "associated" is used instead of "correlated", and "might moderate" were kept to introduce the concepts.

Bibliography

Aulbach, M. B., Bamberg, C., Reichenberger, J., Arend, A.-K., & Blechert, J. (2025). Bidirectional associations between affect and food craving within and between individuals: A mega-analysis. *Appetite*, 107936. https://doi.org/10.1016/j.appet.2025.107936

Bucher Della Torre, S., Carrard, I., Farina, E., Danuser, B., & Kruseman, M. (2017). Development and Evaluation of e-CA, an Electronic Mobile-Based Food Record. *Nutrients*, *9*(1), 76. https://doi.org/10.3390/nu9010076

Colaizzi, J. M., Flagel, S. B., Joyner, M. A., Gearhardt, A. N., Stewart, J. L., & Paulus, M. P. (2020). Mapping sign-tracking and goal-tracking onto human behaviors. *Neuroscience and Biobehavioral Reviews*, *111*, 84–94. https://doi.org/10.1016/j.neubiorev.2020.01.018

Daw, N. D., Gershman, S. J., Seymour, B., Dayan, P., & Dolan, R. J. (2011). Model-based influences on humans' choices and striatal prediction errors. *Neuron*, *69*(6), 1204–1215. https://doi.org/10.1016/j.neuron.2011.02.027

Farr, O. M., & Mantzoros, C. S. (2018). Obese individuals with type 2 diabetes demonstrate decreased

activation of the salience-related insula and increased activation of the emotion/salience-related amygdala to visual food cues compared to non-obese individuals with diabetes: A preliminary study. *Diabetes, Obesity & Metabolism*, *20*(10), 2500–2503. https://doi.org/10.1111/dom.13403

Leys, C., Ley, C., Klein, O., Bernard, P., & Licata, L. (2013). Detecting outliers: Do not use standard deviation around the mean, use absolute deviation around the median. *Journal of Experimental Social Psychology*, *49*(4), 764–766. https://doi.org/10.1016/j.jesp.2013.03.013

Makaronidis, J. M., & Batterham, R. L. (2018). Obesity, body weight regulation and the brain: Insights from fMRI. *The British Journal of Radiology*, *91*(1089), 20170910. https://doi.org/10.1259/bjr.20170910

Maugeri, A., & Barchitta, M. (2019). A Systematic Review of Ecological Momentary Assessment of Diet: Implications and Perspectives for Nutritional Epidemiology. *Nutrients*, *11*(11), Article 11. https://doi.org/10.3390/nu11112696

Najberg, H., Mouthon, M., Coppin, G., & Spierer, L. (2023). Reduction in sugar drink valuation and consumption with gamified executive control training. *Scientific Reports*, *13*(1), 10659. https://doi.org/10.1038/s41598-023-36859-x

Najberg, H., Rigamonti, M., Mouthon, M., & Spierer, L. (2021). Modifying food items valuation and weight with gamified executive control training. *Royal Society Open Science*, *8*(5), 191288. https://doi.org/10.1098/rsos.191288

Pearce, A. L., Fuchs, B. A., & Keller, K. L. (2022). The role of reinforcement learning and value-based decision-making frameworks in understanding food choice and eating behaviors. *Frontiers in Nutrition*, *9*, 1021868. https://doi.org/10.3389/fnut.2022.1021868

Reents, J., & Pedersen, A. (2021). Differences in Food Craving in Individuals With Obesity With and Without Binge Eating Disorder. *Frontiers in Psychology*, *12*, 660880. https://doi.org/10.3389/fpsyg.2021.660880

Roefs, A., Boh, B., Spanakis, G., Nederkoorn, C., Lemmens, L. H. J. M., & Jansen, A. (2019). Food craving in daily life: Comparison of overweight and normal-weight participants with ecological momentary assessment. *Journal of Human Nutrition and Dietetics: The Official Journal of the British Dietetic Association*, *32*(6), 765–774. https://doi.org/10.1111/jhn.12693

Schad, D. J., Rapp, M. A., Garbusow, M., Nebe, S., Sebold, M., Obst, E., Sommer, C., Deserno, L., Rabovsky, M., Friedel, E., Romanczuk-Seiferth, N., Wittchen, H.-U., Zimmermann, U. S., Walter, H., Sterzer, P., Smolka, M. N., Schlagenhauf, F., Heinz, A., Dayan, P., & Huys, Q. J. M. (2020). Dissociating neural learning signals in human sign- and goal-trackers. *Nature Human Behaviour*, *4*(2), 201–214. https://doi.org/10.1038/s41562-019-0765-5

Stice, E., Burger, K. S., & Yokum, S. (2015). Reward Region Responsivity Predicts Future Weight Gain and Moderating Effects of the TaqIA Allele. *The Journal of Neuroscience*, *35*(28), 10316–10324. https://doi.org/10.1523/JNEUROSCI.3607-14.2015

van Alebeek, H., Röttger, M., Kahveci, S., Blechert, J., & Aulbach, M. B. (2024). The Only Constant is Change: Stable vs. Variable Aspects of Food Approach Bias Relate Differently to Food Craving and Intake. *Appetite*, 107726. https://doi.org/10.1016/j.appet.2024.107726

Voon, V., Derbyshire, K., Rück, C., Irvine, M. A., Worbe, Y., Enander, J., Schreiber, L. R. N., Gillan, C., Fineberg, N. A., Sahakian, B. J., Robbins, T. W., Harrison, N. A., Wood, J., Daw, N. D., Dayan, P., Grant, J. E., & Bullmore, E. T. (2015). Disorders of compulsivity: A common bias towards learning habits. *Molecular Psychiatry*, *20*(3), Article 3. https://doi.org/10.1038/mp.2014.44

Voon, V., Reiter, A., Sebold, M., & Groman, S. (2017). Model-Based Control in Dimensional Psychiatry. *Biological Psychiatry*, *82*(6), 391–400. https://doi.org/10.1016/j.biopsych.2017.04.006

Zech, H. G., Gable, P., van Dijk, W. W., & van Dillen, L. F. (2023). Test-retest reliability of a smartphone-based approach-avoidance task: Effects of retest period, stimulus type, and demographics. *Behavior Research Methods*, *55*(5), 2652–2668. https://doi.org/10.3758/s13428-022-01920-6

Answers to reviews

Our reply to each of the comment are in green, with the main modification in the manuscript reported in blue.

Reviewer #1:
Remarks to the Author:
The MS is very ripe now. I recomment acceptance pending minor revisions.

The authors provide a rationale for why implicitly measured cravings might be more strongly associated with learning biases. However, since they formulate a non-directional hypothesis, it would be helpful if they could also explain why learning biases might instead be more strongly associated with explicitly measured cravings.

We added the following sentence (section 3.5.5) to provide a rationale for the non-directional hypothesis: "However, although approach bias may reflect one automatic component of craving, namely a higher tendency to approach food-related stimuli, the learning biases may be associated with other components of cravings (e.g., habit, influence of environmental and physiological cues), leading to stronger associations with the overall subjective experience of craving than with approach bias alone."

Please note that the paper cited by the authors for the AAT procedure has since been retracted. However, another paper describes the same study and therefore the same procedure: https://doi.org/10.1016/j.appet.2026.108462

We thank the reviewer for pointing this out. The reference has been corrected throughout the manuscript.

Reviewer #2:
Remarks to the Author:
This is a very well executed study. I focused on the blue (new parts) of the report. I think the authors did a very good job of being very nuanced in their conclusions so I do not have any major points, only a few minor points:

We thank the reviewer for their positive assessment of the manuscript. The abstract has been revised to improve accessibility by providing additional context and clarifying key concepts.

It would further be important to add some of the main possible causes for the null effects to the abstract including the healthy sample and the floor effect on the consumption measure.

The abstract was revised to include those suggestions: "The hypothesis linking cravings to consumption was not tested due to a consumption floor effect, and neither learning bias was associated with approach bias. The healthy-weight sample may explain our null findings, as they may show limited expression of high-caloric food cravings and consumption. Overall, these findings suggest that reinforcement learning tendencies may contribute to everyday craving dynamics."

Why is the pilot study omitted from the current manuscript?
The pilot analysis has been re-included in the Supplementary Material with mention of it in the main manuscript section 4.2.1: "We conducted a pilot analysis to assess the feasibility and interpretability of the measures used in this study, which can be found in the Supplementary Material."

This argument requires some additional elaboration. "Due to the floor effect observed for H2 (See Supplementary Material for detailed analysis), the final sample target was revised to match that of H1 and H3.

Recruitment continued until the post-exclusion sample included 63 participants with complete datasets. Distribution outliers were not planned to be replaced to avoid circularly changing the thresholds for exclusion."

I understand that it is in the supplements, but still it would be good to explain this a little in the main text. Specifically, when was descried that there is a floor effect and that additional sampling would not address this?

An explanation was added in the Sampling plan (section 3.4): "Due to a floor effect observed for H2 during recruitment, we conducted a floor effect analysis to assess whether further recruitment would mitigate this issue. This indicated that the floor effect would persist despite increasing the sample size (see Section 4.2)." Moreover, as suggested by the editor, we integrated the floor effect analysis in the main manuscript.

Table 2 can be replaced by text.

The table was replaced by text: "Participants had a mean age of 25.8 years (SD = 7.6) and a mean body mass index (BMI) of 22.3 kg/m² (SD = 2.1). The sample comprised 55 women and 26 men (female-to-male ratio = 0.68). The mean questionnaire completion rate was 73.1% (SD = 23.2), and the mean mobile approach-avoidance task (mobile-AAT) completion rate was 69.3% (SD = 28.8)."

In the conclusion it is stated: "In contrast, the positive association between model-free bias and craving provides preliminary evidence for the role of habit-like reinforcement processes in everyday eating." Since there is no action at all in the consumption measure I would rephrase the everyday eating part.
This term was replaced by: "everyday craving experiences."

Maybe reflect on the limitations of Bayes factors for quantifying null findings.
We thank the reviewer for this comment. Bayes factors were included only as a complementary measure of evidence and were not planned to be interpreted. This was made clearer in the Results section 5: "All results were reported alongside Bayes factors as complementary information. However, these were not used as a basis for interpretation."

Reviewer #3:
Remarks to the Author:
I have no objections to the publication of this manuscript.

We thank the reviewer for their positive assessment.

Reviewer #4:
Remarks to the Author:
I co-reviewed this manuscript with one of the reviewers who provided the listed reports. This is part of the Communications Psychology initiative to facilitate training in peer review and to provide appropriate recognition for Early Career Researchers who co-review manuscripts.

We thank the reviewer for their positive assessment.
