# Supplementary material for: The role of affective learning biases in daily food behaviour
Source: Commun Psychol. 2026 Jul 24;4:136. doi: 10.1038/s44271-026-00511-x (PMC13652808; doi:10.1038/s44271-026-00511-x)
Supplement: Supplementary file 2 [file 44271_2026_511_MOESM2_ESM.pdf]

Supplementary Material for Stage 2 RR: “The role of affective learning biases in daily food behaviour”

1. Stimuli

1.1 Sequential learning

The stimuli of the task are the same as in[1] and consisted in boats and monsters on sea and islands backgrounds. All images have been generated using the AI image generator tool Midjourney.

Boat prompt: “Two similar wooden boats standing straight next to each other in the same ocean, exact same size and orientation, one is blue and the other one is white, cartoon style.”

Monsters prompt: “Two cute cartoon rainforest monsters, smiling, in different colours, standing completely straight, distant from each other, same size, looking at us, lilo et stitch style.”

Desert monsters: “In the same cartoon style as (Rainforest monsters image) two cute cartoon rainforest monsters, smiling, in different colours, standing completely straight, distant from each other, same size, looking at us”

After generating different sets of images using variations of these prompts, two boats and four monsters with similar styles and no obvious positive or negative connotation were selected.

Images were then edited using Photoshop and AI clean up tools to simplify the monsters’ and the boats appearance.

Backgrounds were extracted from generated images of rainforest monsters, desert monsters and boats and edited to remove the monsters and boats, as well as most of the original features.

Backgrounds for reward and no reward were generated using DALL·E 2, with the prompt “collection of interiors of a bank safe, one in warm colours, the other one is in cold grey colours in cartoon style”. Images were then simplified using AI clean up tools and colours were adjusted using Photoshop.

1.2 Pavlovian conditioning and Pavlovian-to-Instrumental transfer

The stimuli of the task are the same as in[1]. Crystals and fractals images were generated using the AI tools DALL·E 2 and Stable Diffusion.

Prompts for fractals: “Collection of beautiful fractals in different colours.”

Prompts for crystals: “Collection of beautiful crystals in different colours.”

After generating different sets of images using variations of these prompts, six crystals and five fractals with no obvious positive or negative connotation were selected.

The reward image was chosen from Pixabay with the keyword “money bag” (Pixabay, n.d., https://www.pixabay.com)

1.3 Food icons for EMA self-reported craving and consumption questionnaire

The following icons were uploaded from the Flaticon website with their corresponding attributions:

Biscuit: Biscuit icons created by Freepik - Flaticon

Burger: Burger icons created by Stockio - Flaticon

Cereal: Rice icons created by Freepik - Flaticon

More: Add icons created by Pixel perfect - Flaticon

Pasta: Pasta icons created by Smashicons - Flaticon

Pastry: Croissant icons created by Freepik - Flaticon

Rice: Rice icons created by Freepik - Flaticon

Salad: Vegetable icons created by afif fudin - Flaticon

Soup: Healthy food icons created by Mihimihi - Flaticon¨

All other food icons come from the Microsoft Powerpoint built-in icons library.

2. Pavlovian conditioning computations

To avoid a visual salience effect on the eye gaze at CS presentation, the gaze index is calculated using the last second of CS presentation.

The gaze index is computed as the proportion of fixation time on CS minus the proportion of fixation time on the US location: gaze index = p(CS) – p(US).

A linear regression is then performed for each participant on the gaze index with the true value of CS (-2 CHF, -1 CHF, 0 CHF, 1 CHF, 2 CHF) for each participant.

The computed regression coefficient is then used as a moderator of the intervention efficacy. A positive regression coefficient signifies a gaze attracted more to win-predictive than to loss-predictive CSs and thus corresponds to a sign-tracker tendency, whereas a negative coefficient signifies a gaze attracted towards the goal for expected wins more than for expected losses and expresses a goal-tracker tendency.

3. Sequential learning computations

To evaluate the model-free/model-based bias, we used the reinforcement learning computational modelling as in [2,3]. The algorithm used was the same as in [1], details of the computation can be found below.

The tasks include three stages, first stage being s_A; second stage being s_B or s_C. The action from first stage to second stage is denoted a_A and action between second stage to result (reward or no reward) is denoted a_B.

The objective is to learn the long-run value $Q_{TD}(s, a)$, for each stage s and action a). In a given trial t, the initial state s_A is denoted as $s_{1,t}$, while the subsequent state is noted as $s_{2,t}$. The actions taken in the first and second stages are referred to as $a_{1,t}$ and $a_{2,t}$, respectively, and the corresponding rewards are identified as $r_{1,t}$ (which is always zero) and $r_{2,t}$ (which is =0.5 CHF or 0 CHF).

Model-free learning

The model-free learning is modelled through the SARSA (λ) temporal differences TD algorithm. During each stage *i* of a given trial *t*, the value associated with the state-action pair that was visited is updated according to:

(1) $Q_{TD}(s_{i,t}, a_{i,t}) = Q_{TD}(s_{i,t}, a_{i,t}) + \alpha_i \delta_{i,t}$

where

(2) $\delta_{i,t} = r_{i,t} + Q_{TD}(s_{i+1,t}, a_{i+1,t}) - Q_{TD}(s_{i,t}, a_{i,t})$

To update the value associated with the state-action pair in each stage of a given trial, the expressions first incorporate the value of the resulting stage 2 state, $Q_{TD}(s_{2,t}, a_{2,t})$, in order to update the stage-1 action value. Since no reward is received at this stage, the reward $r_{1,t}$ is set to 0. After that, the stage-2 value is updated considering the reward $r_{2,t}$, and the terminal value $Q_{TD}(s_{3,t}, a_{3,t})$, which is defined as 0. Each stage has its own learning rate parameter (α_1, α_2), which is used to update the value associated with that stage. Additionally, the first-stage action value is updated again using the stage-2 prediction error at the end of each trial:

(3) $Q_{TD}(s_{1,t}, a_{1,t}) = Q_{TD}(s_{1,t}, a_{1,t}) + \alpha_1 \lambda \delta_{2,t}$

The eligibility trace parameter λ dictates the extent of this update.

Model-based learning

In the model-based reinforcement learning algorithm, the first stage action value (Q_{MB}) takes into account the probability of this action leading to s_B or s_C with $P(s_B|s_A, a_A) = 0.7$ and $P(s_C|s_A, a_A) = 0.3$, or vice versa $P(s_B|s_A, a_A) = 0.3$ and $P(s_C|s_A, a_A) = 0.7$, as well as the values of those states. The second stage (where immediate reward is offered) is modelled through the temporal difference TD algorithm, as the estimated value of action does not require further anticipation. Thus, for each action a_j ($j = A, B$):

(4) $Q_{MB}(s_A, a_j) = P(s_B|s_A, a_j) \max_k Q_{TD}(s_B, a_k) + P(s_C|s_A, a_j) \max_k Q_{TD}(s_C, a_k)$

To connect both values, the net value of action of the first choice is computed as a weighted combination of Q_{TD} and Q_{MB}:

(5) $Q_{net}(s_A, a_j) = wQ_{MB}(s_A, a_j) + (1 - w)Q_{TD}(s_A, a_j)$

In which w is the weighting parameter expressing a model-free or model-based biais (w =0 indicates a model-free reliance and w =1 indicates a model-based reliance).
The net value of action of the second choice $Q_{net} = Q_{MB} = Q_{TD}$

Lastly, we employ the softmax equation in Q_{net} to determine the probability of a choice at each stage:

(6) $P(a_{i,t} = a|s_{i,t}) \propto \exp(\beta_i[Q_{net}(s_{i,t}, a) + p * rep(a)])$

The primary outcome of this task is thus the weighting parameter w, which will be used to evaluate how the affective learning bias moderates the intervention's efficacy.

The free parameters will be estimated using a Maximum A Posteriori (MAP) estimation. Priors will be set as follows: beta1 and beta2 will be drawn from a gamma distribution, p from a normal distribution, and alpha1, alpha2, w, and lambda will be drawn from a logit transform of a normal distribution. Details on the parameters estimation and parameter recovery analysis can be found in our previous work[1].

Parameters estimation

Parameters were estimated using Maximum A Posteriori (MAP) estimation. Priors were set as follows: beta1 and beta2 were drawn from a gamma distribution, p from a normal distribution, and alpha1, alpha2, w, and lambda were drawn from a logit-transformed normal distribution (Supplementary Table 1).

A previous parameter recovery analysis using the same model demonstrated good recovery for all estimated parameters (see[1] for details and results of the analysis).

	**α1**	**α2**	**w**	**β1**	**β2**	**P**	**λ**	**-LP**
mean (n=81)	0.430	0.372	0.461	4.123	2.710	0.141	0.549	235.532
std	0.193	0.268	0.192	1.960	1.188	0.149	0.181	42.508

Supplementary Table S1. *Summary of inferred parameters.*

4. Pilot analysis

Prior to data collection, we conducted a pilot analysis to assess the feasibility and interpretability of the measures used in this protocol.

A summary of participant characteristics is provided in the Supplementary Table 2. We measured the sign-tracking and reinforcement learning biases to determine whether their distributions align with those reported in the literature[2,4,5]. The observed ranges closely match those previously documented[2,6], confirming that our tasks capture individual differences in learning biases.

The pilot data and analysis script can be downloaded on our OSF study page (see Code Availability Statement).

**Mean ±SD**	Participants (n=10)
**Age**	23.7 ± 2.3
**Gender Ratio (F/M)**	0.9 (90/10)
**Sign-tracking bias (regression coefficient)**	0.01 ± 0.10
**Reinforcement learning bias (w)**	0.43 ± 0.15

Supplementary Table 2*: Summary of participants' characteristics on pilot data*

5. More items categorisation

Food items entered via the "more" icon were reclassified into HCHP, other, or non-food rather than on the exact category label.

Newly created HCHP categories included sodas and high-calorie dairy products (e.g., cream cheese, ice cream). Newly created non-HCHP categories included low-calorie dairy products (e.g., cottage cheese), eggs, healthy nuts (e.g., pecans and walnuts), and other carbohydrate sources (e.g., legumes and chestnuts). In addition, we recorded certain entries as non-food items, such as alcohol and beverages like black coffee or tea; these were treated as missing (i.e., non-entries) in the analyses.

Categorisation was performed independently by three researchers. For the consensus process, we focused on whether each item was classified as HCHP rather than on the exact category label (e.g., whether an item was labeled as "candy" or "chocolate" was irrelevant, as both are HCHP). Possible categories were defined as HCHP, other, or non-food. Items that did not achieve a two-thirds agreement were further discussed until a final classification was reached. Across the 471 "more" items reported by participants, 79.2% achieved full consensus,

18.1% reached a two-out-of-three agreement, and 2.7% required discussion due to lack of agreement before a final consensus was established.

6. Sample size evolution

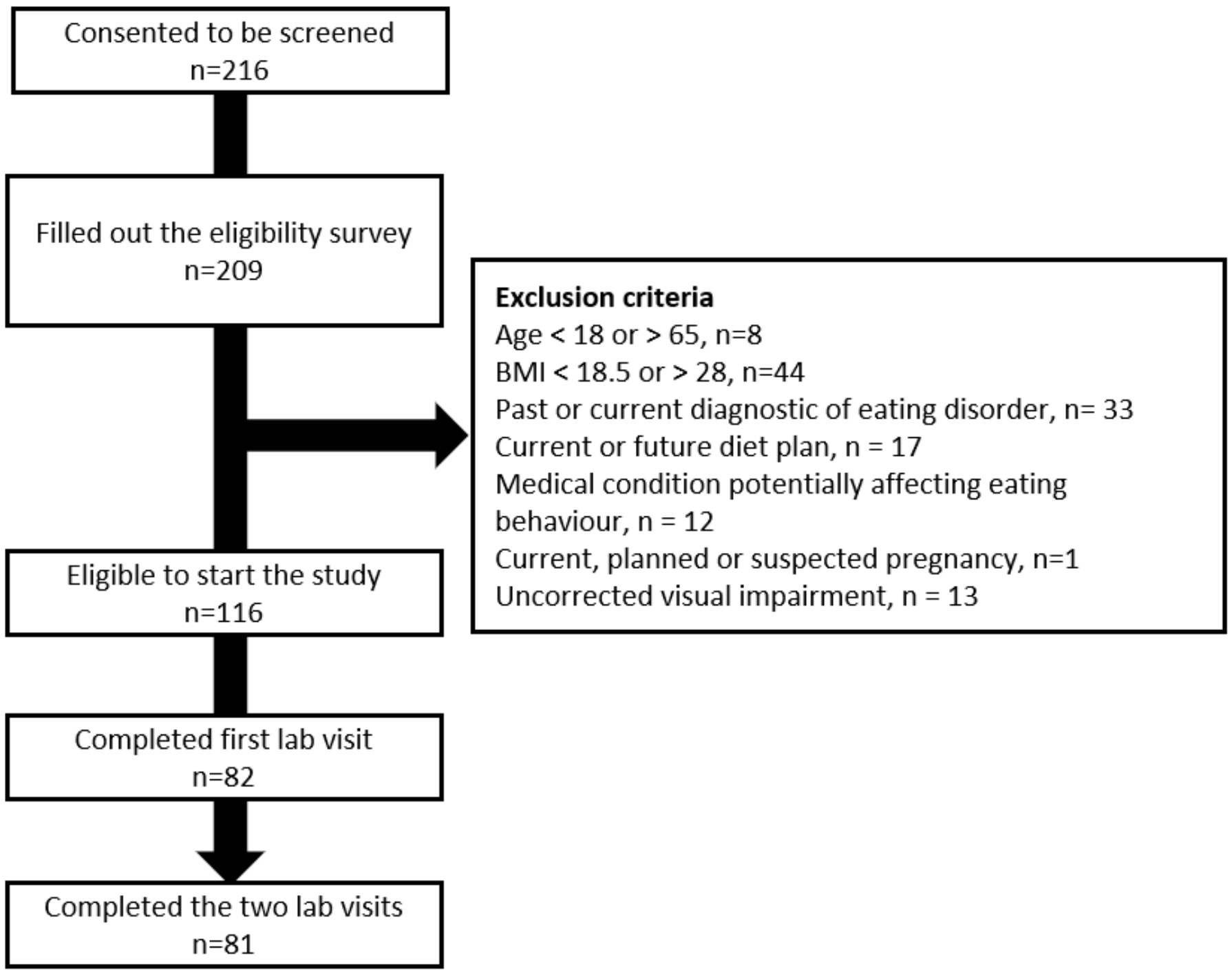

Supplementary Figure 1. *Sample size progression*.

7. Exploratory analysis

7.1 H2a: Learning biases as Pavlovian bias

Exclusion criteria were the same as those used in H1a, resulting in a sample of 69 participants. Ten Pavlovian bias outliers were removed, as in H1a, and one additional participant was excluded due to an outlier in mean consumption. The final sample, therefore, consisted of 58 participants.

A linear mixed-effects model examined the association between daily HCHP consumption and daily HCHP cravings, Pavlovian bias, and their interaction. Daily consumption was entered as the dependent variable, with daily cravings, Pavlovian bias, and their interaction specified as fixed effects. The subject was included as a random intercept to account for repeated observations within individuals. There was no evidence for a significant effect of interaction between craving and Pavlovian bias on consumption (β = 1.39, SE = 0.82, 95% CI [-0.31, 2.91], t(46.96) = 1.58, p = .121), *p* = 0.121), indicating that Pavlovian bias did not moderate the relationship between cravings and consumption. Neither the main effect of craving nor the main effect of Pavlovian bias reached statistical significance (Supplementary figure 2a). The interaction term explained almost no variance in HCHP consumption (Δ marginal R^2 =0.017, full vs no interaction model). A Bayes factor comparing the full model (including the interaction) to the model without the interaction yielded BF_{10} = 1.83, indicating weak evidence for a moderation effect.

7.2 H2b: Learning bias as reinforcement-learning bias w

Exclusion criteria were identical to those applied in H1b, resulting in a sample of 65 participants. No outliers were identified for the bias w, three outliers were identified for the mean cravings and 8 for the mean consumption. The final sample, therefore, consisted of 57 participants.

A linear mixed-effects model tested whether the balance between model-based and model-free control (*w*) moderated the association between daily HCHP cravings and daily HCHP consumption. The interaction term between craving and *w* was not significant (β = -0.09, 95% CI [-0.35, 0.17], SE = 0.13, *t*(44.45) = -0.69, *p* = 0.501), indicating that *w* did not modulate the relationship between cravings and consumption. Neither the main effect of craving nor the main effect of *w* reached statistical significance (Figure 6b). The interaction term explained almost no variance in HCHP consumption (Δ marginal R^2 =0.018, full vs no interaction model). A Bayes factor comparing the full model (including the interaction) to the model without the interaction yielded BF_{10} = 0.55, indicating weak evidence against a moderation effect.

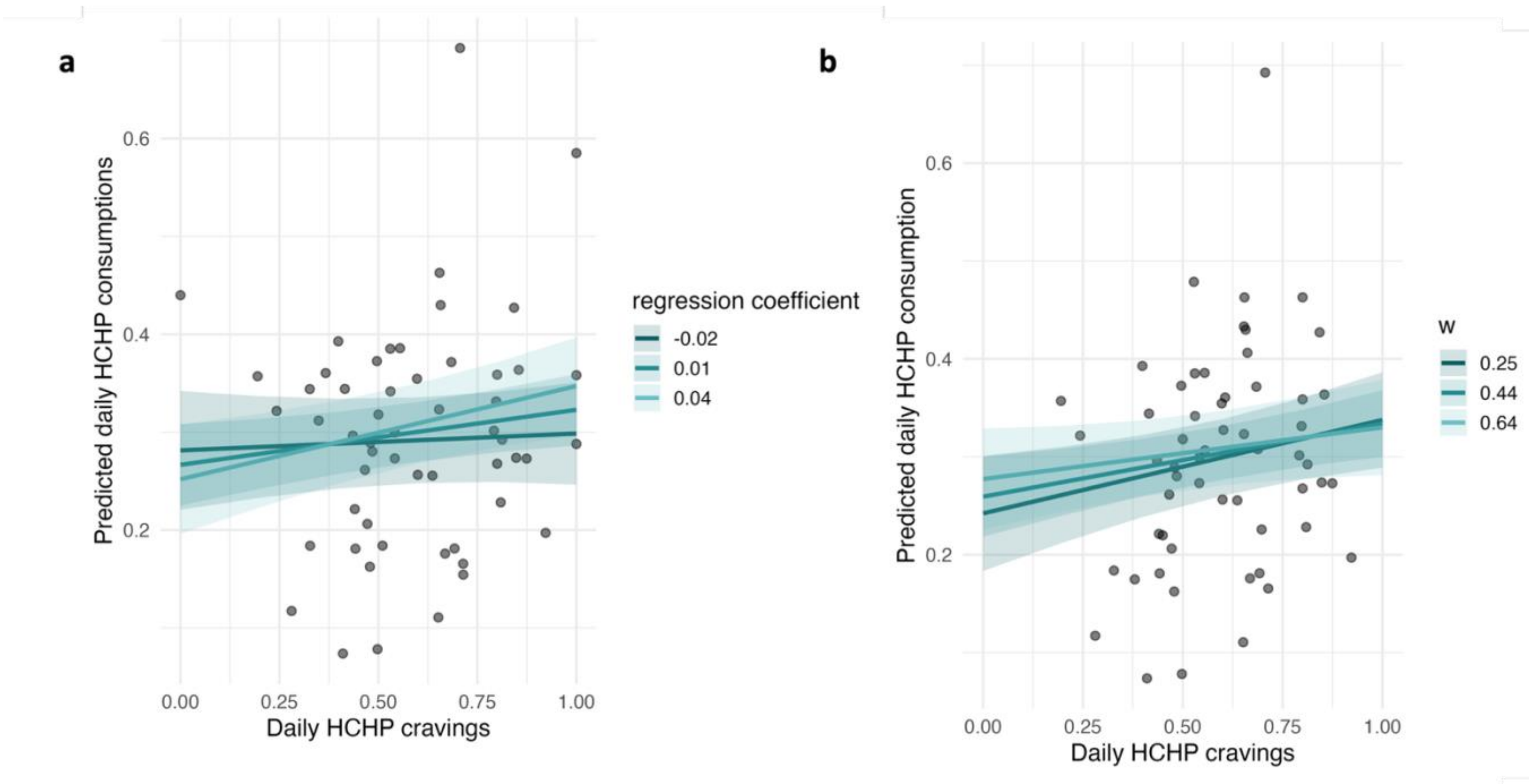

Supplementary Figure 2. *Interaction plots illustrating the relationship between daily HCHP cravings and* model-predicted *daily HCHP consumption. Panel (a) shows predictions at low (-1 SD), mean, and high (+1 SD) levels of Pavlovian bias; panel (b) shows the same predictions for bias* w. *Lines represent fixed-effect predictions, points indicate participant-level mean values, and shaded areas denote 95% confidence intervals around the predicted means.*

7.3 Correlation comparison

For the Pavlovian learning bias, correlations with both self-reported cravings and approach bias were close to zero, yielding a minimal difference between measures ($\Delta r = 0.051$) (Supplementary Figure 3a). Similarly, correlations with variability were close to zero or very small, resulting in only a negligible difference ($\Delta r = 0.096$). Both differences were well below the preregistered threshold of 0.20, indicating no meaningful advantage of either measure in capturing the relationship between Pavlovian bias and craving-related outcomes (Supplementary Figure 2).

For the bias related to sequential learning, the absolute difference between correlations exceeded the 0.20 threshold ($\Delta r = 0.347$), although there was a change in direction preventing interpretation in line with the hypothesis that one measure is a better indicator of the same underlying process (Supplementary Figure 2). For the standard deviation, the difference was below the preregistered threshold ($\Delta r = 0.17$) (Figure 9b).

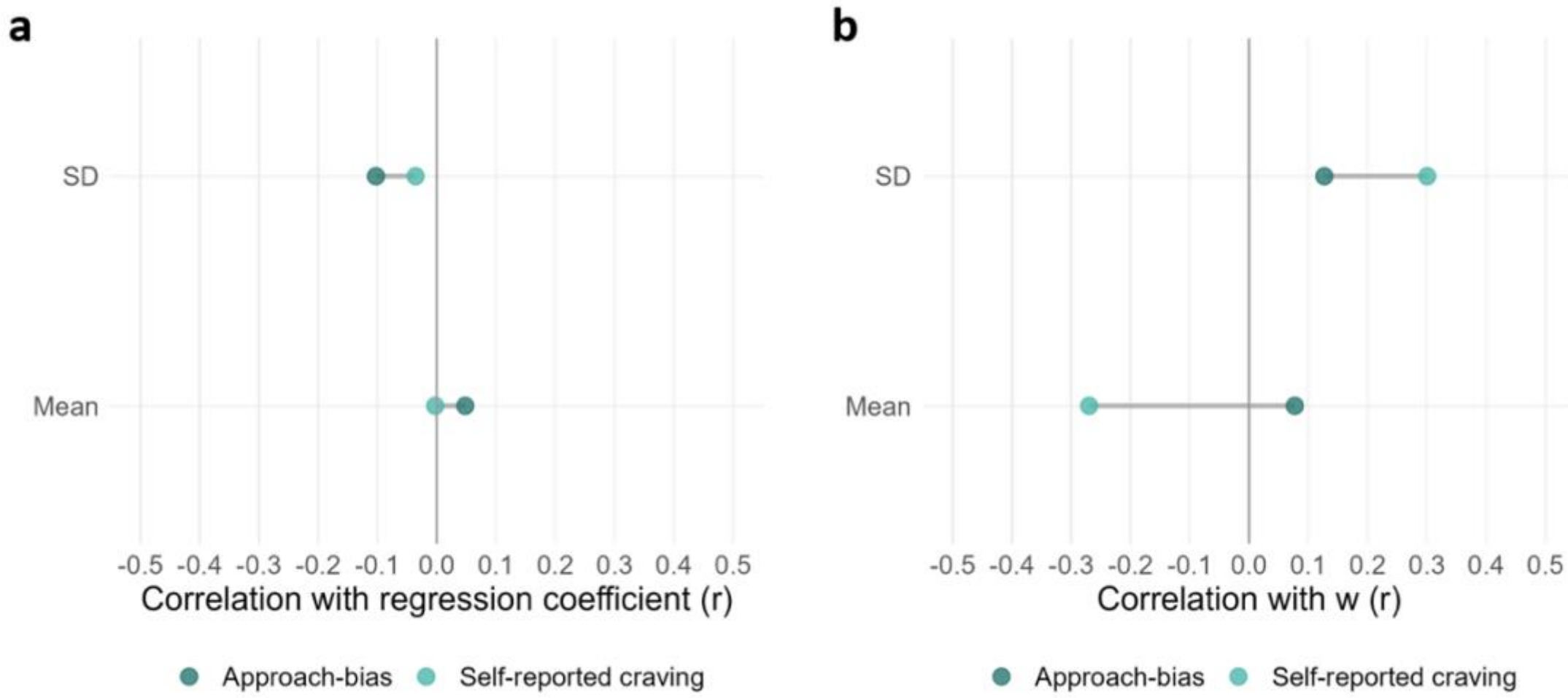

Supplementary Figure 3*. Representation of correlations between learning biases and daily craving metrics measured via self-report and approach-bias. (a) shows correlations between the Pavlovian learning bias (regression coefficient) and two indices of craving/approach bias across the two-week period: mean level and standard deviation (SD). (b) shows the same correlations for the w index. For each outcome, the two points represent correlations with approach-bias and self-reported craving***,*** connected by a line to facilitate comparison. The vertical grey line indicates r = 0.*

8. References

1. Tapparel, M., Pool, E. R., Najberg, H. & Spierer, L. Plastic brain mechanisms supporting reduction in cravings induced by response training. *Imaging Neurosci.* **4**, IMAG.a.1180 (2026) doi:10.1162/IMAG.a.1180.
2. Voon, V., Derbyshire, K., Rück, C., Irvine, M. A., Worbe, Y., Enander, J., Schreiber, L. R. N., Gillan, C., Fineberg, N. A., Sahakian, B. J., Robbins, T. W., Harrison, N. A., Wood, J., Daw, N. D., Dayan, P., Grant, J. E. & Bullmore, E. T. Disorders of compulsivity: a common bias towards learning habits. *Mol. Psychiatry* **20**, 345–352 (2015) doi:10.1038/mp.2014.44.
3. Daw, N. D., Gershman, S. J., Seymour, B., Dayan, P. & Dolan, R. J. Model-Based Influences on Humans’ Choices and Striatal Prediction Errors. *Neuron* **69**, 1204–1215 (2011) doi:10.1016/j.neuron.2011.02.027.
4. Schad, D. J., Garbusow, M., Friedel, E., Sommer, C., Sebold, M., Hägele, C., Bernhardt, N., Nebe, S., Kuitunen-Paul, S., Liu, S., Eichmann, U., Beck, A., Wittchen, H.-U., Walter, H., Sterzer, P., Zimmermann, U. S., Smolka, M. N., Schlagenhauf, F., Huys, Q. J. M., Heinz, A. & Rapp, M. A. Neural correlates of instrumental responding in the context of alcohol-related cues index disorder severity and relapse risk. *Eur. Arch. Psychiatry Clin. Neurosci.* **269**, 295–308 (2019) doi:10.1007/s00406-017-0860-4.

5. Daw, N. D., Gershman, S. J., Seymour, B., Dayan, P. & Dolan, R. J. Model-based influences on humans' choices and striatal prediction errors. *Neuron* **69**, 1204–1215 (2011) doi:10.1016/j.neuron.2011.02.027.
6. Schad, D. J., Rapp, M. A., Garbusow, M., Nebe, S., Sebold, M., Obst, E., Sommer, C., Deserno, L., Rabovsky, M., Friedel, E., Romanczuk-Seiferth, N., Wittchen, H.-U., Zimmermann, U. S., Walter, H., Sterzer, P., Smolka, M. N., Schlagenhauf, F., Heinz, A., Dayan, P. & Huys, Q. J. M. Dissociating neural learning signals in human sign- and goal-trackers. *Nat. Hum. Behav.* **4**, 201–214 (2020) doi:10.1038/s41562-019-0765-5.
